# Reducing the impact of Auger recombination in quasi-2D perovskite light-emitting diodes

**DOI:** 10.1038/s41467-020-20555-9

**Published:** 2021-01-12

**Authors:** Yuanzhi Jiang, Minghuan Cui, Saisai Li, Changjiu Sun, Yanmin Huang, Junli Wei, Li Zhang, Mei Lv, Chaochao Qin, Yufang Liu, Mingjian Yuan

**Affiliations:** 1 Key Laboratory of Advanced Energy Materials Chemistry (Ministry of Education), Renewable Energy Conversion and Storage Center (RECAST), College of Chemistry, Nankai University, 300071 Tianjin, P. R. China; 2 Henan Key Laboratory of Infrared Materials and Spectrum Measures and Applications, College of Physics and Materials Science, Henan Normal University, 453007 Xinxiang, P. R. China

**Keywords:** Energy, Lasers, LEDs and light sources

## Abstract

Rapid Auger recombination represents an important challenge faced by quasi-2D perovskites, which induces resulting perovskite light-emitting diodes’ (PeLEDs) efficiency roll-off. In principle, Auger recombination rate is proportional to materials’ exciton binding energy (*E*_b_). Thus, Auger recombination can be suppressed by reducing the corresponding materials’ *E*_b_. Here, a polar molecule, *p*-fluorophenethylammonium, is employed to generate quasi-2D perovskites with reduced *E*_b_. Recombination kinetics reveal the Auger recombination rate does decrease to one-order-of magnitude lower compared to its PEA^+^ analogues. After effective passivation, nonradiative recombination is greatly suppressed, which enables resulting films to exhibit outstanding photoluminescence quantum yields in a broad range of excitation density. We herein demonstrate the very efficient PeLEDs with a peak external quantum efficiency of 20.36%. More importantly, devices exhibit a record luminance of 82,480 cd m^−2^ due to the suppressed efficiency roll-off, which represent one of the brightest visible PeLEDs yet.

## Abstract (web summary)

Designing efficient perovskite light-emitting diodes remains a challenge due to the strong Auger recombination and resulting Joule heating. Here, the authors propose polarizable *p*-fluorophenethylammonium to generate quasi-2D perovskites with reduced binding energy developing perovskite light-emitting diodes with a peak EQE of 20.36% and a maximum luminance of 82,480 cdm^-2^.

## Introduction

Quasi-2D perovskites with self-assembled multiple-quantum-well structures represent an important category of perovskites, have achieved great success in perovskite light-emitting diodes (PeLEDs)^1–10^. Impressive external quantum efficiencies (EQEs) up to 21.6% in the near-infrared (NIR) region^11^ and 9.5% in the blue region^12^ have been accomplished. Quasi-2D PeLEDs exhibit high EQEs particularly at low injected current densities offering great potential for next-generation display application^13–15^. Unfortunately, quasi-2D PeLEDs suffer from severe efficiency roll-off, which manifests that EQEs start to significantly drop at relatively low current density. Corresponding reasons can be attributed to the strong Auger recombination^16–19^. Efficiency roll-off is a key challenge for quasi-2D PeLEDs, which limits their achievable brightness and thereby impedes the commercialization. The problem thus urgently requires a remedy.

**Fig. 1:**
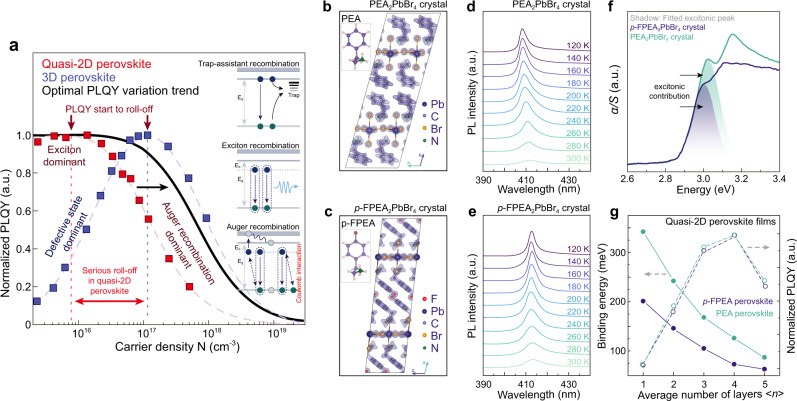
Characteristics of perovskite single crystals and films. **a** Experimental PLQYs of quasi-2D perovskite and 3D perovskite films as a function of carrier density; the curves indicates that serious Auger recombination takes place in quasi-2D case compared to 3D one, leading to rapid PLQY declining at a relatively lower threshold of carrier density; the black curve illustrates an ideal PLQY evolution trend for the optimal perovskite emitter which we want to achieve. Lattice structure and corresponding simulated total electron charge density for **b** PEA_2_PbBr_4_ and **c** *p*-FPEA_2_PbBr_4_, respectively. **d**, **e** Temperature-dependent PL spectra and **f** absorption spectra of PEA_2_PbBr_4_ and *p*-FPEA_2_PbBr_4_ single crystals. **g** Extracted *E*_b_ and corresponding PLQYs for PEA and *p*-FPEA based quasi-2D films with different <*n* > -values; the PLQYs of the films were obtained under low carrier densities of around 1.2 × 10^15^ cm^−3^.

The emission behavior of quasi-2D perovskites is determined by their recombination kinetics. Quasi-2D perovskite is a strongly confined system comprising both quantum- and dielectric confinement, which leads to the formation of strongly bound excitons^13,20^. In quasi-2D films, the amplified carrier density is created at the recombination center due to the efficient energy transfer. Accordingly, shallow defects can be progressively saturated; then first-order exciton recombination outperforms the defect trapping process, lead to high photoluminescence quantum yields (PLQYs)^13^. Basically, the excitonic feature and efficient energy transfer account for the two primary reasons to guarantee the high PLQY, particularly under weak excitation (Fig. 1a).

Unfortunately, the threshold is quite low for Auger recombination to become dominated in quasi-2D perovskites. The reason can attribute to the following reasons. First, the recombination center’s carrier density is orders of magnitude higher compared to 3D perovskite, owing to the aforementioned energy transfer^21,22^. As well known, Auger recombination rate is proportional to the cube of carrier density; hence, the amplified carrier density leads to enhanced Auger recombination. Second, in principle, rapid Auger recombination associates with high exciton binding energy (*E*_b_) because of the enhanced Coulomb electron–hole interaction^23–25^. The enhanced interaction leads to carriers no longer uniformly distributed in space, thus enlarge the probability of finding two electrons and one hole at the same position to accelerate the Auger process (Supplementary Fig. 1)^24–27^. In practice, Auger recombination rate is proportional to the third power of the *E*_b_ in strongly confined 1D material^28^. Accordingly, quasi-2D perovskites should exhibit fast Auger recombination because of their large *E*_b_. These two characteristics thus enable rapid Auger recombination to happen in quasi-2D films.

Recently, increasing attention has been attracted to address this detrimental effect. For instance, Wang et al. demonstrated a composition engineering approach to increase the amounts of recombination center in quasi-2D films^16^. The resulting decreased carrier density slowed down the Auger process and suppressed the efficiency roll-off. However, the problem has yet to be fully solved due to the limited number of solutions. We noticed structure engineering was a promising way to suppress Auger recombination, which has achieved great success in quantum dots and nanowires area^29–31^. Manipulation of dielectric confinement to reduce the electron–hole wavefunction overlap is considered as the most powerful approach in quantum dots research^32–34^. However, structure engineering innovation has yet been tentatively explored in quasi-2D perovskites to tailor Auger recombination.

Auger recombination rate is proportional to the third power of *E*_b_, in strongly confined 1D material^28^. Thus, reducing *E*_b_ can suppress Auger recombination in 1D material. Accordingly, we conceive to explore whether reducing *E*_b_ could also alter the Auger recombination rates in quasi-2D perovskites. However, developing a good emitter is not as simple as that. In brief, reducing *E*_b_ also will decrease first-order exciton recombination, which is against to achieve high PLQY, especially under low excitation density. This is because the film’s PLQY is determined by the compromise between exciton recombination and trap-assisted nonradiative recombination under weak excitation^13,26^. Consequently, reducing trap-assisted recombination rates is highly required to minimize the negative effects induced by the decreased *E*_b_. On the other hand, decreased *E*_b_ by simply increasing *<n* > -values is not an ideal approach, since increasing *<n* > -values usually leads to inefficient energy transfer^26^. Instead, manipulation of organic cations to weaken the “dielectric confinement” seems to be a better approach, since the approach can notably reduce *E*_b_ without altering energy transfer efficiency.

Here we show, reduction of *E*_b_ is enabled by introducing high-polar organic cation, *p*-fluorophenethylammonium (*p*-FPEA^+^), into the “A-site” of the quasi-2D perovskites. Corresponding *p*-FPEA_2_MA_*n*-1_Pb_*n*_Br_3*n*+1_ perovskite exhibits several times smaller *E*_b_ compared to its phenethylammonium (PEA^+^) analog. As expected, Auger recombination constant determined to be more than one-order-of-magnitude lower. Nevertheless, the first-order exciton recombination rate also decreases meanwhile, which leads to a notable PLQY decline that is unwanted for LED application. Molecular passivation then is applied to suppress the trapping process. Consequently, significantly reduced trap-assistant recombination enables the film’s PLQY to become independent with the decreased exciton recombination. The resulting films exhibit high and invariant PLQYs in a broad range of excitation density. We then are able to demonstrate one of the most efficient green PeLEDs to date with the peak EQE of 20.36%^35^. Moreover, due to the slow efficiency roll-off, the devices exhibit a record luminance of 82,480 cd m^−2^, representing one of the brightest visible PeLEDs yet (EQE > 15%)^4,5,36–38^. Suppressed Auger recombination also reduces the resulting Joule heating, thus notably enhances stability, especially under high luminance. The innovation here paves the way for regulating Auger recombination of quasi-2D perovskite optoelectronics.

## Results

### Manipulation of exciton binding energy

Quasi-2D Ruddlesden–Popper perovskites possess a general formula of (RNH_3_)_2_(A)_*n*-1_B_*n*_X_3*n*+1_, where *R* represents an aromatic or alkyl moiety^39,40^. The *n*-values stand for the number of inorganic [BX_6_] octahedral layers sandwiched between the organic barriers. Quasi-2D perovskites possess a quantum-well structure, and the resulting excitons are confined within the inorganic slabs. In addition, beyond the quantum-confinement, dielectric confinement also arises, which is induced by the dielectric constant mismatch between inorganic well and surrounding organic ligands^41^. Basically, the surrounding organic ligands with small dielectric constants are less polar, thus decrease the dielectric screening of electron–hole Coulomb interaction, which induces the dielectric confinement. As a result, *E*_b_ is additionally strengthened by the dielectric confinement in quasi-2D perovskites. For example, *E*_b_ was determined to be as high as 470 meV for BA_2_PbI_4_ perovskite^42^. Hence, it is possible to decrease *E*_b_ by weakening the dielectric confinement. In principle, increasing the dielectric constant of organic cations can weaken the dielectric-confinement and then result in decreased *E*_b_^42,43^.

PEA_2_MA_*n*−1_Pb_*n*_Br_3*n*+1_ perovskite is a classical quasi-2D material widely used in PeLEDs. In order to inherit its extraordinary emitting property, the framework of this perovskite must be kept; we thus used highly polarized *p*-FPEA^+^ to replace traditional PEA^+^ at “A-site” to regulate *E*_b_. Compared to PEA^+^, the hydrogen atom at *para*-position of phenyl group is substituted by a fluorine atom to generate *p*-FPEA^+^. The presence of an electron-withdrawing fluorine atom would polarize the electronic state of *p*-FPEA^+^ to induce a strong molecule dipole moment^44,45^. Density function theory (DFT) is employed to simulate this dipole moment. The values are determined to be 2.39 D and 1.28 D for *p*-FPEA^+^ and PEA^+^, respectively. Increased dipole moment facilitates the charge separation, leading to increased dielectric constants. Consequently, decreased dielectric constant mismatch diminishes the *E*_b_ in corresponding *p*-FPEA_2_MA_*n*−1_Pb_*n*_Br_3*n*+1_ perovskite. Polarized *p*-FPEA^+^ cation has been used to prepare pure 2D perovskite materials previously, and preliminary studies demonstrated good optical properties^46–50^.

To precisely evaluate the variation trend of *E*_b_, we grew the single crystal of *n* = 1 PEA_2_PbBr_4_ and *p*-FPEA_2_PbBr_4_ perovskite for investigation. The corresponding lattice structure is depicted in Fig. 1b, c and Supplementary Fig. 2; detailed crystallographic data for *p*-FPEA_2_PbBr_4_ is described in Supplementary Table 2. As shown in Fig. 1f, the optical absorption spectra can be characterized by an excitonic peak at lower energies and an extended absorption edge representing to the band-to-band electronic transitions. Particularly, we resolved the low-energy excitonic contribution in the optical absorption spectra for every single crystal^51^. As expected, an obvious excitonic absorption peak could be observed at around 3.08 eV for PEA_2_PbBr_4_, demonstrating a pronounced excitonic resonance^52,53^. In contrast, the excitonic contribution only displays a kink at around 3.04 eV for *p*-FPEA_2_PbBr_4_, undoubtedly indicating the decreased exciton binding energy^54^. Thus, we can qualitatively deduce a reduced *E*_b_ for *p*-FPEA_2_PbBr_4_ perovskite.

To further strengthen the conclusion, we conducted temperature-dependent photoluminescence (PL) measurements to quantitatively extract the *E*_b_ (Fig. 1d, e). The spectra featured with PL intensity reduction and spectral line broadening, with increased temperature. The extracted *E*_b_ is estimated to be 347 and 195 meV for PEA_2_PbBr_4_ and *p*-FPEA_2_PbBr_4_, respectively (Supplementary Fig. 3)^55^. The results reconfirm that the electron-withdrawing fluorine group in *p*-FPEA^+^ dose decrease the *E*_b_, due to the reduced dielectric confinement. It is worth mentioning that, the perovskite materials undergo a slight structural transition with decreasing temperature (Supplementary Figs. 2 and 3)^41^.

Different *<n* > -values quasi-2D perovskite films were fabricated through a single-step spin-coating process with the help of antisolvents (“<*n* >” represents a quasi-2D domain)^6^. The *<n* >-values can be well controlled by adjusting ratios between different precursors (Supplementary Figs. 4–8 and Supplementary Tables 1, 3). We extracted the *E*_b_ for each of the perovskite films with different *<n* > -values from temperature-dependent PL measurements (Supplementary Figs. 9 and 10). As expected, *p*-FPEA-based quasi-2D perovskites continuously exhibit several times smaller *E*_b_ than the PEA one in each *<n* > -value, confirming the organic cations can effectively modulate *E*_b_ (Fig. 1g).

Outstanding film’s PLQY is the prerequisite for highly performed LED devices. We thus recorded the PLQY evolution as a function of the film’s *<n* > -values to select the most suitable *<n* > -values for further investigation. As shown in Fig. 1g, maximum PLQY is found for the *<n* > = 4 films in both *p*-FPEA and PEA perovskites, which is consistent with the previous results^2^. The high PLQY is proved to be related to the efficiency of energy transfer. In specific, *<n* > = 4 films possess a better-graded energy landscape compared to lower *<n* > -values, which facilitate the energy transfer^21^. On the other hand, when *<n* > -values are beyond 4, the energy landscape at the recombination center becomes flat, which blocks the energy transfer pathway and leads to the PLQY decline^22^ (Supplementary Figs. 6–8 and Supplementary Note 1). Nevertheless, *<n* > = 4 film is selected for further investigation due to its outstanding emission property.

### Recombination dynamics of perovskite films with different *E*_b_

**Fig. 2:**
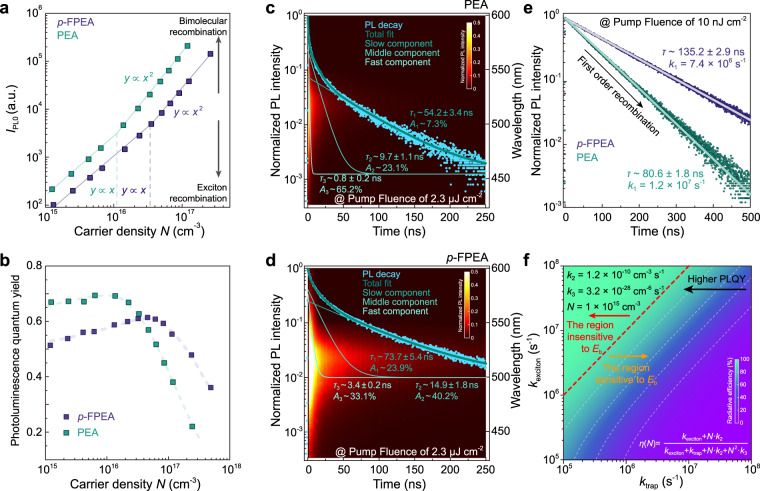
Recombination dynamics of quasi-2D films with different *E*_b_. **a** PL intensity at *t* = 0 (*I*_PL0_) as a function of carrier density. **b** Film’s PLQYs as a function of carrier density for PEA and *p*-FPEA quasi-2D films, respectively. Time- and wavelength-dependent photoluminescence (PL) mapping under a pump fluence of 2.3 μJ cm^−2^ for **c** PEA and **d** *p*-FPEA films. **e** TRPL decay transients for PEA and *p*-FPEA perovskite films under a low pump fluence of 10 nJ cm^−2^. **f** Simulated radiative efficiency as a function of *k*_exciton_ and *k*_trap_ for quasi-2D films.

Carrier recombination mechanism is highly essential for understanding materials emission behavior. We first investigated the PL intensity at *t* = 0 (*I*_PL0_), at the instant of the pulse excitation, as the function of excitation density for *p*-FPEA and PEA films (Fig. 2a and Supplementary Fig. 11a, b). Scaling of *I*_PL0_ with excitation density is a widely used tool to uncover the nature of recombination^56^. As shown in Fig. 2a, a clear transition from linear to super-linear dependent *I*_PL0_ is observed with increased excitation density for both samples. In specific, *I*_PL0_ is linear with excitation density under low fluence (~10^16^ cm^−3^ for PEA film and ~4 × 10^16^ cm^−3^ for *p*-FPEA film, respectively), indicating the characteristics of exciton recombination (monomolecular process). Afterward, a quadratic dependence *I*_PL0_ with excitation density arises at high excitation density, which is consistent with the bimolecular recombination feature. The phenomenon reveals that the excitons formed in *<n* > = 4 films are likely to dissociate to free carriers when excitation density goes up, in good agreement with the previous reports^5^.

According to the above recombination mechanism, under steady-state excitation, the theoretical radiative emission quantum yield $\eta (N)$ for quasi-2D perovskite can be given by the well-known equation^13^:

1 $$\eta \left( N \right) = \frac{{k_{{\mathrm{exciton}}} + N\cdot k_2}}{{k_{{\mathrm{exciton}}} + k_{{\mathrm{trap}}} + N\cdot k_2 + N^2\cdot k_3}}$$

where *N* is the carrier density, *k*_exciton_ is the first-order radiative exciton recombination constant, *k*_trap_ is the monomolecular trap-assistant recombination constant, *k*_2_ is the bimolecular recombination constant, and *k*_3_ is the three-body Auger recombination constant, respectively. As shown, the PLQY is strongly dependent on *N*, illustrating the compromise between first-order recombination (containing both excitonic and trap-assistant process), bimolecular recombination, and Auger recombination. In specific, PLQY depends on the competition between first-order exciton recombination and trap-assistant nonradiative recombination under low excitation intensity. When increasing the *N*, radiative bimolecular recombination gradually dominates over the monomolecular process. At even higher carrier density, three-body Auger recombination becomes very effective and dominant, resulting in PLQY decline.

To qualitatively analyze the recombination kinetics, we tracked the film’s PLQY variation as a function of carrier density for both samples, as shown in Fig. 2b. As expected, we did find the higher threshold for PLQY declining in *p*-FPEA film, illustrating the Auger recombination constant (*k*_3_) decreased. However, we noticed the PLQY of *p*-FPEA film dropped around 20% at low carrier density compared to the PEA one. The phenomenon is attributed to the decreased *E*_b_ as well since the exciton recombination constants (*k*_exciton_) are also proportional to the *E*_b_ in quantum wells^25^. According to Eq. (1), when the trap-assistant recombination constant (*k*_trap_) is non-negligible, the film’s PLQY decreases with reduced *k*_exciton_.

We then applied time-resolved photoluminescence (TRPL) measurements to extract the rate constants of monomolecular, bimolecular, and trimolecular recombination^56^. The PL decay traces under different carrier densities are shown in Supplementary Fig. 12. Under low fluences, the PL decays follow single-exponential behavior, which is consistent with the characteristics of first-order exciton recombination. When increasing the fluences to a higher level, some fast decays appear in the PL dynamics, then gradually become dominant. With increased excitation density, the amplitude of these fast component increases, and the resulting effective lifetime decreases (Supplementary Fig. 11c). These fast decay components are ascribed to the combination of bimolecular radiative and trimolecular Auger recombination^16^. Accordingly, multi-exponential fitting is applied to analyze the carrier dynamics under high excitation (Fig. 2c, d)^57^. The corresponding carrier density (*N*) is estimated to be as high as ~1 × 10^17^ cm^−3^, warranting trimolecular Auger recombination to take place. The fastest decay component is then assigned to Auger recombination; interestingly, the component would fall back to a single-exponential slope at longer times, because of the diminished carrier density^56^. We fitted a fast decay time of 0.8 ns to the Auger recombination in PEA sample; While the fast decay time is elongated to 3.4 ns in the *p*-FPEA case under the same excitation density. The data qualitatively reveal that Auger recombination rate is notably decreased after *p*-FPEA substitution.

Moreover, carrier dynamics can be quantitatively described by the following equation according to the recombination mechanism^13^:

2 $$\frac{{\mathrm{d}}N (t)}{{{\mathrm{d}}}t} = - k_3\cdot N^3 - k_2\cdot N^2 - k_1\cdot N$$

We employed numerical integration of Eq. (2) to simultaneously simulate all the kinetics for each sample. To constrain the fitting, only *k*_2_ and *k*_3_ were set as free fitting parameters. The first-order recombination constant was then experimentally determined from the TRPL kinetics under low pump fluences, where the first-order recombination was dominant and the high-order recombination contribution was negligible (Supplementary Table 4). As shown in Fig. 2e, the fitted first-order recombination constants, *k*_1_, are determined to be 1.2 × 10^7^ s^−1^ and 7.4 × 10^6^ s^−1^ for PEA and *p*-FPEA, respectively. Afterward, transient absorption measurement with various pump fluences was conducted (Supplementary Fig. 13). The bleach kinetics under different pump fluences are analyzed through a global fitting procedure to simultaneously simulate the data. The global fitting reveals that the bimolecular and trimolecular recombination constants are decreased for *p*-FPEA perovskite, as displayed in Table 1. It is worth mentioning that the trimolecular Auger recombination constants are more than one-order-of magnitude lower for *p*-FPEA case when compared to the PEA analog, reconfirming that the Auger recombination rate has been notably suppressed through *p*-FPEA substitution, and in good agreement with the above TRPL analysis.

**Table 1:** PLQYs (*η*_PL_) under carrier density of ~1.2 × 10^15^ and ~2.3 × 10^17^ cm^−3^, monomolecular (*k*_1_), bimolecular (*k*_2_), and trimolecular (*k*_3_) recombination constant, estimated defect state density (*N*_t_) and hole mobility (*μ*) of different quasi-2D perovskite films.

	*η*_PL_^†^	*η*_PL_^‡^	*k*_1_ (s^−1^)	*k*_2_ (cm^3^ s^−1^)	*k*_3_ (cm^6^ s^−1^)	*N*_t_ (cm^−3^)^§^	*μ* (cm^2^ V^−1^ s^−1^)^§^
PEA	0.66	0.22	1.2(±0.1) × 10^7^	8.7 (±1.0) × 10^−10^	7.9 (±2.1) × 10^−27^	2.6 × 10^16^	0.8 × 10^−3^
*p*-FPEA	0.51	0.48	7.4(±0.4) × 10^6^	1.2 (±0.4) × 10^−10^	3.2 (±0.9) × 10^−28^	2.1 × 10^16^	2.2 × 10^−3^
CF_3_KO_3_S-PEA	0.85	0.30	1.1(±0.1) × 10^7^	9.1 (±0.9) × 10^−10^	8.3 (±3.2) × 10^−27^	1.7 × 10^15^	1.2 × 10^−3^
CF_3_KO_3_S-*p*-FPEA	0.82	0.55	5.3(±0.2) × 10^6^	1.4 (±0.3) × 10^−10^	3.6 (±0.8) × 10^−28^	1.3 × 10^15^	3.2 × 10^−3^

^†^Measured under the carrier density of ~1.2 × 10^15^ cm^−3^.

^‡^Measured under the carrier density of ~2.3 × 10^17^ cm^−3^.

^§^Extracted from SCLC measurements.

Although the Auger recombination constant is suppressed, the corresponding PLQY especially at low excitation density is notably reduced, which is unwanted for LED application (Fig. 2b). As shown, first-order recombination constants, *k*_1_, reduces from 1.2 × 10^7^ s^−1^ and 7.4 × 10^6^ s^−1^ after PEA substituted by *p*-FPEA. However, we could not quantitatively distinguish between exciton recombination constants (*k*_exciton_) and trap-assistant nonradiative recombination constants (*k*_trap_) from the global fitting, because both of them possess monomolecular recombination feature^12^. However, we could qualitatively conclude that the *k*_exciton_ does decrease after the *p*-FPEA substitution, by comparing the initial PL intensity (*I*_PL0_) shown in Fig. 2a^5^. According to Eq. (1), the PLQY would drop significantly with decreased *k*_1_, when the *k*_trap_ is non-negligible. Fortunately, according to the equation, PLQY would become insensitive to the *k*_exciton_, if the *k*_trap_ can be reduced to several times or one-order-of magnitude lower level compared to *k*_exciton_. To confirm this hypothesis, we simulated the radiative efficiency as a function of *k*_exciton_ and *k*_trap_ under the carrier density of 10^15^ cm^−3^, according to the equation. As reflected in Fig. 2f, as expected, the PLQYs do reach a high level and become independent with *k*_exciton_, once the *k*_trap_ is one-order-of magnitude lower than *k*_exciton_. Accordingly, exploring an effective passivation method to decrease the *k*_trap_ is thus important to minimize the negative effect of *E*_b_ reduction.

### Minimizing the trap-assistant recombination

**Fig. 3:**
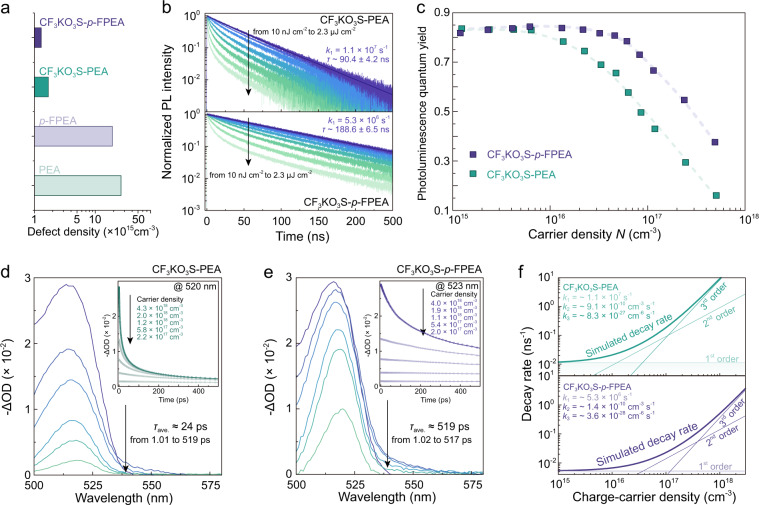
Recombination dynamics for CF_3_KO_3_S treated quasi-2D films. **a** Extracted defects density for different perovskite films according to SCLC measurements. **b** TRPL spectra for CF_3_KO_3_S-PEA and CF_3_KO_3_S-*p*-FPEA films under different excitation. **c** Film’s PLQYs as a function of carrier density for CF_3_KO_3_S-PEA and CF_3_KO_3_S-*p*-FPEA quasi-2D films, respectively. TA spectra at different delay time under carrier density of ~4 × 10^18^ cm^−3^ for **d** CF_3_KO_3_S-PEA and **e** CF_3_KO_3_S-*p*-FPEA films; the insets are the bleaching kinetics with different fluences. **f** Simulated carrier decay rates as a function of carrier density for CF_3_KO_3_S-PEA and CF_3_KO_3_S-*p*-FPEA films.

In spite of the shallow defects that can be saturated by energy transfer, deep-level defects still exist and need to be carefully treated. It was demonstrated that deep-level defects of perovskites mainly derived from the halide vacancy and under-coordinated lead atoms^58,59^. Organic anions are considered as effective passivation reagents, that bound with exposed under-coordinated metal atoms and filled halide vacancy. Accordingly, Lewis base-metal adduct, potassium trifluoromethanesulfonate (CF_3_KO_3_S), was introduced to passivate the defects especially the deep-level defects^60,61^. We employed space-charge-limited current (SCLC) techniques to evaluate the defect state density of the quasi-2D films before and after CF_3_KO_3_S treatment (Supplementary Fig. 14). The onset voltage of the trap-filled-limit (*V*_TFL_) region is proportional to the density of defect states^57^. The extracted defect densities are found to decrease to one-order-of magnitude lower after CF_3_KO_3_S passivation (Fig. 3a and Table 1), implying the *k*_trap_ is greatly reduced after passivation.

First-order recombination kinetics are recorded to investigate the variation trend of *k*_trap_. The intensity of *I*_PL0_ as the function of excitation density is conducted to uncover the nature of recombination (Supplementary Fig. 15). As shown, the transition from linear to quadratic-dependent *I*_PL0_ is observed with increased carrier density for both films, illustrating that the photo-generated excitons under weak excitation tend to dissociate to free carriers at higher excitation density. Accordingly, the first-order recombination constant is extracted from TRPL spectra under low carrier density, which is reflected as a single-exponential decay (Fig. 3b and Supplementary Table 4). The fitted first-order recombination rates are determined to be 1.1 × 10^7^ s^−1^ and 5.3 × 10^6^ s^−1^ for CF_3_KO_3_S-PEA and CF_3_KO_3_S-*p*-FPEA films, respectively. In combination with PLQY evolution as a function of carrier density, we can qualitatively distinguish the contribution to overall first-order recombination rates between *k*_trap_ and *k*_exciton_. As shown in Fig. 3c, the PLQYs are quite high and do not exhibit any difference between CF_3_KO_3_S-PEA and CF_3_KO_3_S-*p*-FPEA films under low carrier density, which is completely different from the phenomenon before CF_3_KO_3_S passivation; where the PLQYs of *p*-FPEA films exhibit notable decline compared to the PEA sample. Combining the above findings, we can conclude the *k*_trap_ has been reduced to much a lower level after CF_3_KO_3_S treatment because only negligible *k*_trap_ can enable film PLQY becoming insensitive to *k*_exciton_ declining under low excitation density.

Global fitting was carried out to simultaneously simulate all the kinetic for the treated films, as shown in Fig. 3d, e. Notably, the trimolecular Auger recombination constant, *k*_3_, is confirmed to be more than one-order-of magnitude lower for CF_3_KO_3_S-*p*-FPEA film compared to the CF_3_KO_3_S-PEA one (Table 1). The global fitting suggests that CF_3_KO_3_S treatment does not alter the high-order recombination kinetics, but significantly suppresses the trap-assistant recombination rate. Consequently, the PLQY of CF_3_KO_3_S-*p*-FPEA quasi-2D film displays nearly constant value within a broad region of carrier density, demonstrating the first-order exciton recombination and bimolecular recombination always control over the recombination channel.

Numerical simulation was conducted to evaluate the decay rates as a function of carrier density, using experimentally extracted kinetics as a parameter (Supplementary Figs. 16 and 17). As exhibited in Fig. 3f, the simulation results indicate that the required threshold for trimolecular Auger recombination to dominate the recombination channel should be much higher for CF_3_KO_3_S-*p*-FPEA quasi-2D films than the CF_3_KO_3_S-PEA one. The simulation results are in good agreement with the experiment data. As shown in Fig. 3c, the PLQY for CF_3_KO_3_S-*p*-FPEA keeps constant in a wide range of carrier density^16^. More importantly, the carrier density calculated to be five times larger in CF_3_KO_3_S-*p*-FPEA film than CF_3_KO_3_S-PEA, regarding the threshold of PLQY roll-off. In summary, after CF_3_KO_3_S treatment, the CF_3_KO_3_S-*p*-FPEA films not only possess high and invariant PLQY at low carrier density but also show suppressed Auger recombination at high carrier density, which behaves as an optimal perovskite emitter for LED application (Supplementary Tables 3, 5 and Supplementary Note 2).

### High-performance PeLEDs with suppressed efficiency roll-off

**Fig. 4:**
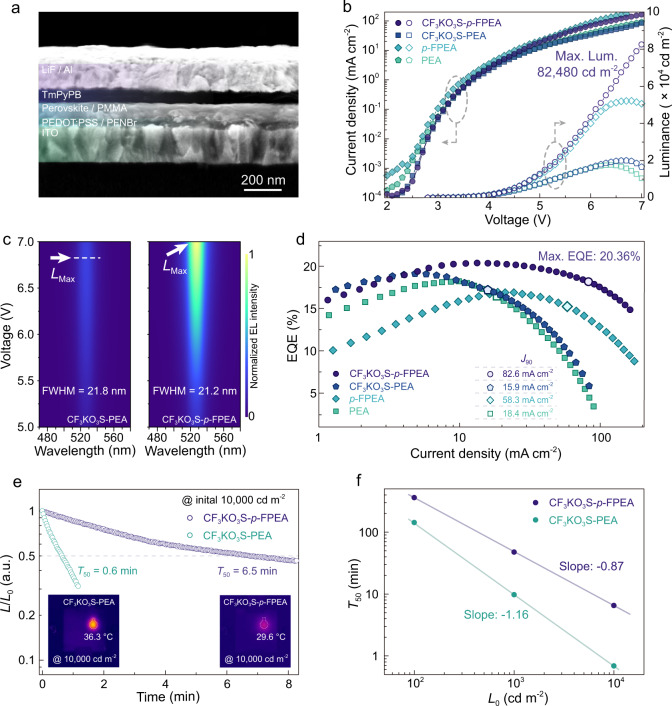
Device performance of the resulting PeLEDs. **a** Cross-section SEM image for CF_3_KO_3_S-*p*-FPEA PeLEDs. **b** *I–V* and *L–V* curves for PeLEDs based on different quasi-2D perovskites. **c** EL spectra as a function of voltage for CF_3_KO_3_S-PEA and CF_3_KO_3_S-*p*-FPEA PeLEDs. **d** Current density-dependent EQE curve for different quasi-2D PeLEDs. **e** Half-lifetime (*T*_50_) measurements and corresponding spatial surface temperature for PeLEDs at an initial luminance of 10,000 cd m^−2^. **f** Half-lifetime (*T*_50_) of PeLEDs at different initial luminance.

We then fabricated PeLEDs with device configuration of ITO/PEDOT:PSS (20 nm)/perovskite (100 nm)/TmPyPB (40 nm)/LiF (0.8 nm)/Al (100 nm), to evaluate the impact of suppressed Auger recombination on device performance. We further introduced PFN-Br and PMMA thin layer at the interface to passivate interfacial defects and facilitate charge inject efficiency (Supplementary Fig. 23)^62^. A cross-section scanning electron microscope (SEM) image is displayed in Fig. 4a. Confocal fluorescence microscopy (CFM) characterization reveals uniform PL emission without any visible defect appeared (Supplementary Fig. 24). Top-view SEM and atomic force microscopy (AFM) images also confirm that the films possessed dense and desirable morphology (Supplementary Figs. 25 and 26). In addition, all the films exhibit extremely low surface roughness (r.m.s.) (<3.1 nm), which is important to prevent leakage current.

As expected, the electroluminescence (EL) performance for *p*-FPEA quasi-2D perovskite is greatly improved under high current densities compared to PEA based devices (Fig. 4b, c). By effectively eliminating the trap-assisted nonradiative recombination, the CF_3_KO_3_S-*p*-FPEA devices exhibit excellent properties, which display high efficiency under a broad range of injected current density (Fig. 4d and Supplementary Table 6). Notably, a peak EQE of 20.36% is achieved for the CF_3_KO_3_S-*p*-FPEA PeLEDs, which represents one of the most efficient green PeLEDs reported to date^35^.

We tracked the dependence of EQE on the injected current density and defined the *J*_90_ (the current density at EQE drops to 90% of the maximum) to quantify the efficiency roll-off (Fig. 4d)^17,63^. As revealed, the *J*_90_ of CF_3_KO_3_S-*p*-FPEA device is determined to be 82.6 mA cm^−2^, which is over five times higher than that of CF_3_KO_3_S-PEA device (15.9 mA cm^−2^). We point out that the EQE evolution trend is in good agreement with PLQYs variation trend under different excitation densities (Fig. 3c), which further confirms the EQE roll-off is mainly induced by the rapid Auger recombination (Supplementary Note 3)^14^. As expected, the CF_3_KO_3_S-*p*-FPEA devices exhibit an extraordinary maximum luminance of 82,480 cd m^−2^ with excellent color purity (Fig. 4c and Supplementary Figs. 27–29. The result represents one of the brightest visible PeLEDs together with high efficiency (EQE > 15%), which is important for display application (Supplementary Table 7)^4,5,36–38^.

Since Auger recombination leads to Joule heating, which significantly impedes device stability under high current density. We then investigated the impact of suppressed Auger recombination on device stability. In particular, we set the initial luminance as 10,000 cd m^−2^ to examine the EL intensity variation. As shown, the CF_3_KO_3_S-PEA device loses 50% of its initial EL intensity within 0.6 min (*T*_50_); while the CF_3_KO_3_S-*p*-FPEA device exhibit a *T*_50_ of 6.5 min, which account for one-order-magnitude improvement (Fig. 4e and Supplementary Fig. 30). In order to uncover the reasons, we monitored the surface temperature of the glass substrate (Fig. 4e and Supplementary Fig. 31). The CF_3_KO_3_S-PEA device exhibits a temperature of 36.3 °C after operated at 10,000 cd m^−2^ for 10 s. In contrast, the highest surface temperature of the CF_3_KO_3_S-*p*-FPEA device can reach about 29.6 °C. Accordingly, we attributed the temperature increase mainly due to the nonradiative Auger recombination^18^. Furthermore, we also recorded the *T*_50_ under different intimal luminance, to fit the acceleration factor (n) according to empirical scaling law *L*_0_^n^·*T*_50_ = constant (Fig. 4f and Supplementary Fig. 32). We found that the trend of operational stability declines with the increasing of initial luminance. Thus, we confirm that the suppressed Auger recombination in perovskite layers plays a critical role for operational stability improvement, particularly at high luminance^63–65^.

## Discussion

Rapid nonradiative Auger recombination rate is an important challenge faced by quasi-2D perovskites, which causes significant efficiency roll-off and impedes their further commercialization. As far as we know, Auger recombination rate is proportional to the materials’ exciton binding energy (*E*_b_); thereby, Auger process might be suppressed by reducing the corresponding *E*_b_ in principle. Accordingly, a polar molecule, *p*-FPEA, is introduced into the lattice to generate quasi-2D perovskites with reduced *E*_b_. Recombination dynamics data reveal the Auger recombination rate does decrease to one-order-of magnitude lower level. Unfortunately, the first-order exciton recombination rate is also decreased meanwhile, leads to a notable PLQY decline which is unwanted for LED application. Hence, molecular passivation agent, CF_3_KO_3_S, is used to reduce trap-assistant recombination rate, which enables the film’s PLQY to become independent with exciton recombination rate decline. The resulting films exhibit high PLQY at a broad range of carrier intensity. We thus are able to report one of the most efficient green PeLEDs to date with the peak EQE of 20.36%. Moreover, due to the slow efficiency roll-off, the device shows a record luminance of 82,480 cd m^−2^, representing one of the brightest visible PeLEDs together with high efficiency to date. Suppressed Auger recombination also reduces the resulting Joule heating, thus notably elongates device stability in particular under high current density. The work paves the way for the development of quasi-2D perovskite optoelectronics in the near future.

## Methods

### Materials

Methylammonium bromide (MABr) was purchased from Dyesol. Phenethylamine (PEA), *p*-fluorophenethylamine (*p*-FPEA), lead (II) bromide (PbBr_2_), lead oxide (PbO), potassium trifluoromethanesulfonate (CF_3_KO_3_S), and polymethyl methacrylate (PMMA) were purchased from Sigma-Aldrich. 1,3,5-tri(m-pyrid-3-yl-phenyl)benzene (TmPyPB), poly[(9,9-bis(3*’*-((*N*,*N*-dimethyl)-*N*-ethylammonium)-propyl)-2,7-fluorene)-alt-2,7-(9,9-dioctylfluorene)] (PFN-Br), lithium fluoride (LiF) were purchased from Lumtech Corp. PEDOT:PSS solution (Clevios P VP Al4083) was purchased from Heraeus. Methylammonium acetate (MAAc; liquid) was purchased from Xi’an Polymer Light Technology Corp., Ltd. All of the reagents were directly used as received.

### Perovskite single crystals

PEAPbBr_4_ and *p*-FPEAPbBr_4_ single crystals were grown by lowering the temperature. Specifically, PbO was first dissolved into a mixed solution of HBr and H_3_PO_2_, and then heated to 120 °C to yield a transparent solution. PEA or *p*-FPEA was added into HBr solution using a separate vial under stirring. The above two solutions were then mixed under 120 °C with stirring, gradually decreased to 50 °C. Then the clear plate-shaped crystals appeared in the solution.

### Device fabrication

PEDOT:PSS layer was spin-coated onto the pre-treated ITO substrate at 3,000 r.p.m. for 60 s, and baked at 150 °C for 20 min under ambient conditions. A thin PFN-Br layer (<5 nm) was then spin-coated on PEDOT:PSS film. Perovskite layers were prepared to follow a single-step procedure with the help of antisolvent (chlorobenzene), as documented (Supplementary Table 1)^6^. On the top of perovskite emission layers, 20 μL PMMA solution (0.5 mg ml^−1^ in chlorobenzene) was spin-coated at 5000 r.p.m. for 60 s. TmPyPB (40 nm), LiF (0.8 nm), and Al electrode were thermally evaporated on the top of the devices with an effective area of 8.57 mm^2^.

### Characterizations of perovskite single-crystal and resulting films

Ultraviolet–vis absorption spectra of single crystals were conducted on Shimadzu UV-2550 (diffuse-reflectance). Transmission and reflection spectra of the perovskite films were recorded using a dual-beam UV–vis–NIR spectrophotometer (Cary 5000, Agilent). The absorption coefficient curves of the films were calculated from transmission and reflection spectra. Top-view and cross-section scanning electron microscope (SEM) images were acquired by field-emission SEM (JSM-7500F, JEOL). Confocal fluorescence microscopy (CFM) images were measured using an LSM 880 laser CFM. Atomic force microscopy (AFM) images were collected by Dimension Icon (Bruker) with noncontact mode. The XRD patterns of perovskite films were recorded using Bruker D8 diffractometer with Cu Kα radiation. Crystallographic data of *p*-FPEAPbBr_4_ single crystal was collected on a Bruker APEX II CCD diffractometer with Mo Kα radiation (50 kV, 30 mA), followed by integrating and scaling with multi-scan absorption correction using SAINT and SADABS. The structure was solved and refined by Patterson maps and SHELXL-2014 (full-matrix least-squares on *F*^2^) program, respectively.

### Steady-state and time-resolved photoluminescence (PL) measurement

Steady-state PL spectra were obtained through a fluorescence spectrophotometer (Fluoromax 4, Horiba) with a 450 W Xe lamp. Time-resolved PL (TRPL) spectra were achieved by using an Edinburgh Instruments spectrometer (FLS980). The excitation source was a picosecond pulsed laser with a pulse width of below 100 ps and a repetition rate of 800 kHz at 355 nm. A time-correlated single-phono counting (TCSPC) system was employed to resolve the PL dynamics; the total instrument response function (IRF) was less than 100 ps. The PLQYs were carried out through a three-step technique by a Quanta-Phi integrating sphere with a Fluorolog system under the excitation wavelength of 365 nm. The PLQYs and TRPL at different excitation intensities were recorded at the same external conditions.

### Temperature-dependent PL measurements

Temperature-dependent PL measurements were conducted on Horiba, LabRAM HR 800 equipped with a liquid-nitrogen-cooled cryostat (Linkam). A 325-nm laser with a power of 3 μW was used to excite the samples.

### Transient absorption (TA) measurement

Broadband femtosecond-TA measurements were performed on a pump–probe system (Helios, Ultrafast System LLC) coupled with an amplified femtosecond laser system (Coherent) under ambient conditions. The pulse beam was excited by a Ti:sapphire regenerative amplifier (Legend Elite-1K-HE; pulse width, 35 fs; pulse energy, 7 mJ per pulse; repetition rate, 1 kHz; 800 nm), and seeded with a mode-locked Ti:sapphire laser system (Mica 5) and an Nd:YLF laser (EvolutIon 30) pumped. This pulse beam was then split into two portions. The larger portion was passed an optical parametric amplifier (TOPAS-800-fs, Coherent) to generated the pump pulse (pulse width, around 100 fs; pulse energy, 18.8 μJ per pulse; repetition rate, 1 kHz; 365 nm). The smaller portion (~0.1 μJ per pulse) of the 800-nm pulse beam was focused into a 1 mm CaF_2_ to produce the white light continuum (WLC) probe pulse (from 380 nm to 600 nm). The pulse-to-pulse fluctuation of the WLC probe pulse was corrected by a reference beam split from itself. The pump and probe beam were focused on an overlapped circular spot on the sample with a diameter of 150 μm. A mechanical chopper operated at a frequency of 500 Hz was used to modulate the pump pulses. The temporal and spectral profiles (chirp-corrected) of the pump-induced differential transmission of the WLC probe light (i.e., absorbance change) were visualized by an optical fiber-coupled multichannel spectrometer (with a CMOS sensor). All the TA data were obtained and averaged from at least five scans to ensure the accuracy and high signal-to-noise ratio necessary for global analysis.

### Device characterizations

PeLED devices were tested by Keithley 2400 source meter coupled with a fiber spectrometer (QE65 Pro, FOIS-1-FL integration sphere). *J–V–L* data were collected under a scanning rate of 0.1 V s^−1^ with a dwell time of 1 s. The performance of PeLEDs was double-checked by a PR-735 spectroradiometer (Photo Research). Angular dependence of emission intensity was carried out on a Thorlabs PDA100A detector. Thermal images of operating PeLEDs were detected from the glass side by a thermal imaging system (Fotric 220, ZXF, USA).

## Supplementary information

Peer Review File

Supplementary Information

## Data Availability

The data that support the finding of this study is available from the corresponding author upon reasonable request. The crystallographic data are available in The Cambridge Crystallographic Data Center (CCDC): *p*-FPEA_2_PbBr_4_: 2006717.

## References

[1] Wang N (2016). Perovskite light-emitting diodes based on solution-processed self-organized multiple quantum wells. Nat. Photonics.

[2] Quan LN (2020). Edge stabilization in reduced-dimensional perovskites. Nat. Commun..

[3] Qin C (2020). Triplet management for efficient perovskite light-emitting diodes. Nat. Photonics.

[4] Yang X (2018). Efficient green light-emitting diodes based on quasi-two-dimensional composition and phase engineered perovskite with surface passivation. Nat. Commun..

[5] Ban M (2018). Solution-processed perovskite light emitting diodes with efficiency exceeding 15% through additive-controlled nanostructure tailoring. Nat. Commun..

[6] Jiang Y (2019). Spectra stable blue perovskite light-emitting diodes. Nat. Commun..

[7] Zhao B (2018). High-efficiency perovskite-polymer bulk heterostructure light-emitting diodes. Nat. Photonics.

[8] Li Z (2019). Modulation of recombination zone position for quasi-two-dimensional blue perovskite light-emitting diodes with efficiency exceeding 5%. Nat. Commun..

[9] Cho H (2015). Overcoming the electroluminescence efficiency limitations of perovskite light-emitting diodes. Science.

[10] Liu, X. et al. Metal halide perovskites for light-emitting diodes. *Nat. Mater*. 10.1038/s41563-020-0784-7 (2020). 10.1038/s41563-020-0784-7 32929252

[11] Xu W (2019). Rational molecular passivation for high-performance perovskite light-emitting diodes. Nat. Photonics.

[12] Liu Y (2019). Efficient blue light-emitting diodes based on quantum-confined bromide perovskite nanostructures. Nat. Photonics.

[13] Xing G (2017). Transcending the slow bimolecular recombination in lead-halide perovskites for electroluminescence. Nat. Commun..

[14] Chen Z (2018). Recombination dynamics study on nanostructured perovskite light-emitting devices. Adv. Mater..

[15] Zou C, Liu Y, Ginger DS, Lin LY (2020). Suppressing efficiency roll-off at high current densities for ultra-bright green perovskite light-emitting diodes. ACS Nano.

[16] Zou W (2018). Minimising efficiency roll-off in high-brightness perovskite light-emitting diodes. Nat. Commun..

[17] Lim J, Park YS, Wu K, Yun HJ, Klimov VI (2018). Droop-free colloidal quantum dot light-emitting diodes. Nano. Lett..

[18] Zhao L, Lee KM, Roh K, Khan S, Rand BP (2019). Improved outcoupling efficiency and stability of perovskite light-emitting diodes using thin emitting layers. Adv. Mater..

[19] Bae WK (2013). Controlling the influence of Auger recombination on the performance of quantum-dot light-emitting diodes. Nat. Commun..

[20] Deng S (2020). Long-range exciton transport and slow annihilation in two-dimensional hybrid perovskites. Nat. Commun..

[21] Yuan M (2016). Perovskite energy funnels for efficient light-emitting diodes. Nat. Nanotechnol..

[22] Quan LN (2017). Tailoring the energy landscape in quasi-2D halide perovskites enables efficient green-light emission. Nano. Lett..

[23] Klimov VI, Mikhailovsky AA, McBranch DW, Leatherdale CA, Bawendi MG (2000). Quantization of multiparticle Auger rates in semiconductor quantum dots. Science.

[24] Hangleiter A, Häcker R (1990). Enhancement of band-to-band Auger recombination by electron-hole correlations. Phys. Rev. Lett..

[25] McGuire JA, Joo J, Pietryga JM, Schaller RD, Klimov VI (2008). New aspects of carrier multiplication in semiconductor nanocrystals. Acc. Chem. Res..

[26] Milot RL (2016). Charge-carrier dynamics in 2D hybrid metal-halide perovskites. Nano. Lett..

[27] Hangleiter A (1993). Recombination of correlated electron-hole pairs in two-dimensional semiconductors. Phys. Rev. B.

[28] Wang F, Wu Y, Hybertsen M, Heinz T (2006). Auger recombination of excitons in one-dimensional systems. Phys. Rev. B.

[29] Shen H (2019). Visible quantum dot light-emitting diodes with simultaneous high brightness and efficiency. Nat. Photonics.

[30] Zhu H (2015). Lead halide perovskite nanowire lasers with low lasing thresholds and high-quality factors. Nat. Mater..

[31] Schlaus AP (2019). How lasing happens in CsPbBr_3_ perovskite nanowires. Nat. Commun..

[32] Hou X (2019). Engineering Auger recombination in colloidal quantum dots via dielectric screening. Nat. Commun..

[33] Wang LW, Califano M, Zunger A, Franceschetti A (2003). Pseudopotential theory of Auger processes in CdSe quantum dots. Phys. Rev. Lett..

[34] Bae WK (2013). Controlled alloying of the core-shell interface in CdSe/CdS quantum dots for suppression of auger recombination. ACS Nano.

[35] Lin K (2018). Perovskite light-emitting diodes with external quantum efficiency exceeding 20 per cent. Nature.

[36] Wu C (2019). Alternative type two-dimensional-three-dimensional lead halide perovskite with inorganic sodium ions as a spacer for high-performance light-emitting diodes. ACS Nano.

[37] Lee S (2018). Control of interface defects for efficient and stable quasi-2D perovskite light-emitting diodes using nickel oxide hole injection layer. Adv. Sci..

[38] Han D (2018). Efficient light-emitting diodes based on in situ fabricated FAPbBr_3_ nanocrystals: the enhancing role of the ligand-assisted reprecipitation process. ACS Nano.

[39] Jiang Y (2018). Reduced-dimensional *α*-CsPbX_3_ perovskites for efficient and stable photovoltaics. Joule.

[40] He T, Jiang Y, Xing X, Yuan M (2020). Structured perovskite light absorbers for efficient and stable photovoltaics. Adv. Mater..

[41] Hong X, Ishihara T, Nurmikko AV (1992). Dielectric confinement effect on excitons in PbI_4_-based layered semiconductors. Phys. Rev. B.

[42] Cheng B (2018). Extremely reduced dielectric confinement in two-dimensional hybrid perovskites with large polar organics. Commun. Phys..

[43] Li X (2019). Two-dimensional Dion-Jacobson hybrid lead iodide perovskites with aromatic diammonium cations. J. Am. Chem. Soc..

[44] Shi J (2019). Fluorinated low-dimensional Ruddlesden-Popper perovskite solar cells with over 17% power conversion efficiency and improved stability. Adv. Mater..

[45] Pan H, Zhao X, Gong X, Shen Y, Wang M (2019). Atomic-Scale Tailoring of organic cation of layered Ruddlesden-Popper perovskite compounds. J. Phys. Chem. Lett..

[46] Papagiannouli I, Maratou E, Koutselas I, Couris S (2014). Synthesis and characterization of the nonlinear optical properties of novel hybrid organic-inorganic semiconductor lead iodide quantum wells and dots. J. Phys. Chem. C..

[47] Vassilakopoulou A, Papadatos D, Koutselas I (2016). Room temperature light emitting diode based on 2D hybrid organic-inorganic low dimensional perovskite semiconductor. Appl. Mater. Today.

[48] Vareli I, Vassilakopoulou A, Koutselas I (2018). Defect variants based on the 2D hybrid organic-inorganic low-dimensional semiconductor (4-Fluoro-phenethylamine-H)_2_PbBr_4_ for fabrication of single-layer deep blue LEDs. ACS Appl. Nano Mater..

[49] Papavassiliou G, Koutselas I, Terzis A, Whangbo M (1994). Structural and electronic properties of the natural quantum-well system (C_6_H_5_CH_2_CH_2_NH_3_)_2_SnI_4_. Solid State Commun..

[50] Xu Z, Mitzi D, Dimitrakopoulos C, Maxcy K (2003). Semiconducting perovskites (2-XC_6_H_4_C_2_H_4_NH_3_)_2_SnI_4_ (X= F, Cl, Br): steric interaction between the organic and inorganic layers. Inorg. Chem..

[51] Stoumpos C (2017). High members of the 2D Ruddlesden-Popper halide perovskites: synthesis, optical properties, and solar cells of (CH3(CH2)3NH3)2(CH3NH3)4Pb5I16. Chem.

[52] Saba M, Quochi F, Mura A, Bongiovanni G (2016). Excited state properties of hybrid perovskites. Acc. Chem. Res..

[53] Ishihara T (1994). Optical properties of PbI-based perovskite structures. J. Lumin..

[54] Cao D, Stoumpos C, Farha O, Hupp J, Kanatzidis M (2015). 2D homologous perovskites as light-absorbing materials for solar cell applications. J. Am. Chem. Soc..

[55] Yuan F (2020). Bright high-colour-purity deep-blue carbon dot light-emitting diodes via efficient edge amination. Nat. Photonics.

[56] Richter JM (2016). Enhancing photoluminescence yields in lead halide perovskites by photon recycling and light out-coupling. Nat. Commun..

[57] Shi D (2015). Low trap-state density and long carrier diffusion in organolead trihalide perovskite single crystals. Science.

[58] Li W (2018). Control of charge recombination in perovskites by oxidation state of halide vacancy. J. Am. Chem. Soc..

[59] Yang D (2019). CsPbBr_3_ quantum dots 2.0: benzenesulfonic acid equivalent ligand awakens complete purification. Adv. Mater..

[60] Nenon DP (2018). Design principles for trap-free CsPbX_3_ nanocrystals: enumerating and eliminating surface halide vacancies with softer Lewis bases. J. Am. Chem. Soc..

[61] Wang H (2019). Trifluoroacetate induced small-grained CsPbBr_3_ perovskite films result in efficient and stable light-emitting devices. Nat. Commun..

[62] Dai X (2014). Solution-processed, high-performance light-emitting diodes based on quantum dots. Nature.

[63] Wang H (2020). Perovskite-molecule composite thin films for efficient and stable light-emitting diodes. Nat. Commun..

[64] Cao Y (2018). Perovskite light-emitting diodes based on spontaneously formed submicrometre-scale structures. Nature.

[65] Zhang Q (2019). Efficient metal halide perovskite light-emitting diodes with significantly improved light extraction on nanophotonic substrates. Nat. Commun..

