# Supplementary material for: Reducing the impact of Auger recombination in quasi-2D perovskite light-emitting diodes
Source: Nat Commun. 2021 Jan 12;12:336. doi: 10.1038/s41467-020-20555-9 (PMC7804015; doi:10.1038/s41467-020-20555-9)
Supplement: Supplementary file 2 — Supplementary Information [file 41467_2020_20555_MOESM2_ESM.pdf]

Supplementary Information

Reducing the Impact of Auger Recombination in Quasi-2D Perovskite Light-Emitting Diodes

Yuanzhi Jiang[1], Minghuan Cui[2], Saisai Li[1], Changjiu Sun[1], Yanmin Huang[1], Junli Wei[1], Li Zhang[1], Mei Lv[1], Chaochao Qin[2,*], Yufang Liu[2] & Mingjian Yuan[1,*]

[1] Key Laboratory of Advanced Energy Materials Chemistry (Ministry of Education), Renewable Energy Conversion and Storage Center (RECAST), College of Chemistry, Nankai University, 300071, Tianjin, P. R. China.

[2] Henan Key Laboratory of Infrared Materials and Spectrum Measures and Applications, Henan Normal University, 453007, Xinxiang, P. R. China.

* E-mail: yuanmj@nankai.edu.cn;

qinchaochao@htu.edu.cn;

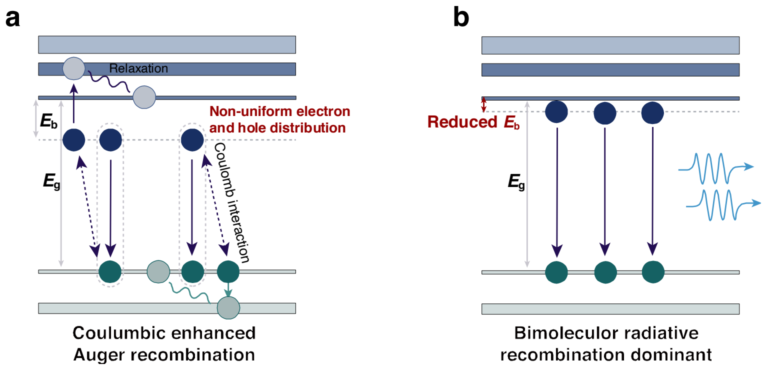

Supplementary Figure 1 | Illustration of charge-carrier recombination processes. Illustration of charge-carrier recombination processes **(a)** with and **(b)** without strong Coulomb interaction between electrons and holes. Since the strong Coulomb interaction between electrons and holes, the electron density in the vicinity of a hole is increased while it is decreased for another electron[1]. Thus, the enhanced Coulomb interactions lead to carriers no longer uniformly distributed in space. Auger recombination rate strongly depends on the particle density, this nonuniform distribution can induce to Coulomb-enhanced Auger recombination (Supplementary Figure 1a). Thus, the Auger recombination rate (k_{Auger}) can be written in the form[1]:

$$k_{\text{Auger}} = g_{eeh}\, k^{0}_{\text{Auger}} = g_{eeh}\, c_n\, n^2 p \tag{1}$$

where k^{0}_{Auger} is the band-to-band Auger recombination rate for noninteracting particles; c_n is the Auger coefficient; n and p are the densities of electrons and holes, respectively; g_{eeh} is the Coulomb enhancement factor, which directly affected by E_b. Keeping this in mind, reducing E_b is a promising strategy to suppress the Coulomb enhancement factor, leading to reduced Auger recombination rates theoretically (Supplementary Figure 1b).

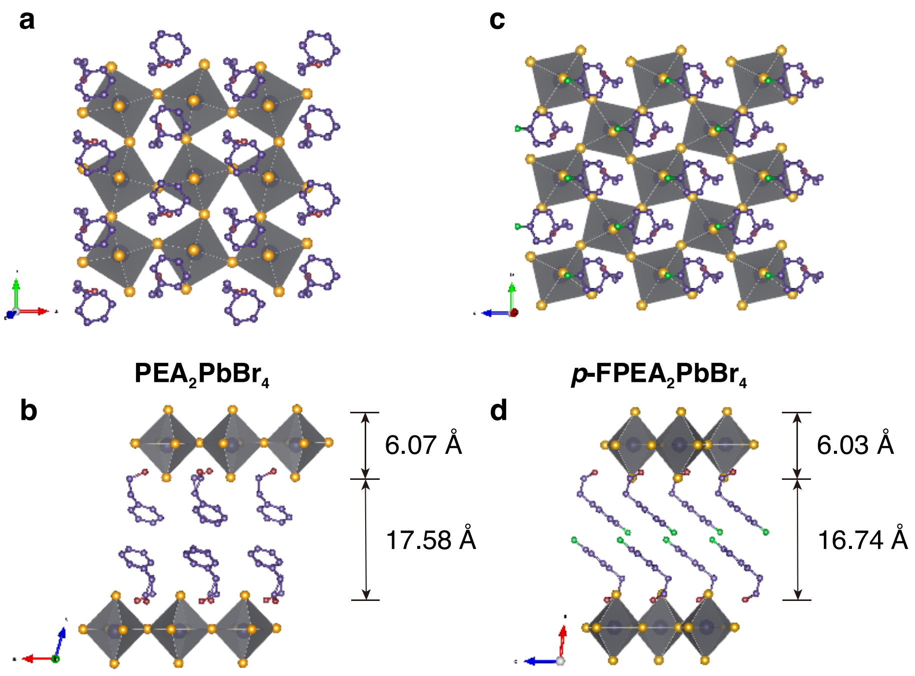

Supplementary Figure 2 | Lattice structures of 2D perovskites. Lattice structures of 2D **(a, b)** PEA_2PbBr_4 and **(c, d)** *p*-$FPEA_2PbBr_4$ perovskites from different view directions. Introducing the fluorine atom on the *para*-position of PEA cation, leads to a change in the packing of organic cations. The packing of *p*-FPEA cations in *p*-$FPEA_2PbBr_4$ perovskite exhibits the same direction and a π-π stacking. The width of the inorganic and organic layers for 2D perovskite is then estimated (Supplementary Figure 2b, d)[2]. The distance of two axial Br ions is measured to be 6.07 Å and 6.03 Å for PEA_2PbBr_4 and *p*-$FPEA_2PbBr_4$. The diameter of a Br ion is 3.92 Å. Thus, the thickness of the inorganic layer is approximately 9.99 and 9.95 Å, respectively. The distance between two inorganic layers is obtained from the lattice constant within crystallographic data. Due to the different packing motifs of PEA^+ and *p*-$FPEA^+$ as well as octahedral distortion, the width of the inorganic and organic layers displays a slight difference. The slightly decreased width of the organic layer within *p*-$FPEA_2PbBr_4$ maybe an additional reason for the reduced E_b[3]. Detailed crystallographic data for *p*-$FPEA_2PbBr_4$ perovskite are provided in Supplementary Table 2.

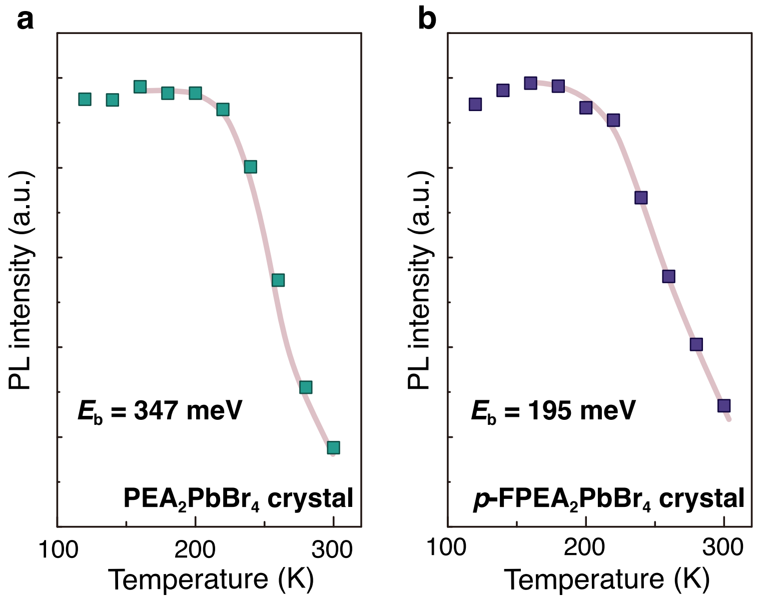

Supplementary Figure 3 | Temperature-dependent integrated photoluminescence (PL) intensity at different temperatures for the 2D perovskite single crystals. Temperature-dependent integrated PL intensity [$I(T)$] at different temperature (T) and corresponding fitting curves for 2D **(a)** PEA_2PbBr_4 and **(b)** p-$FPEA_2PbBr_4$ perovskite single crystals. The perovskite materials generally undergo a slight structural phase transition with the decreasing of the temperature. However, this aspect does not influence the dielectric confinement problem at hand. The exciton binding energy of each perovskite single crystal has been extracted through the fitting procedure.

The exciton binding energies of the perovskite signal crystals and films are evaluated by the temperature dependence of integrated PL signal in terms of the aforementioned method[3]; and the exciton binding energy is fitted by the following equation[4]:

$$I(T) = \frac{I_0}{1 + Ae^{\left(-\frac{E_b}{k_B T}\right)}} \tag{2}$$

where I_0 is the integrated PL intensity extrapolated at 0 K, A is a constant, E_b is the binding energy and k_B is the Boltzmann constant.

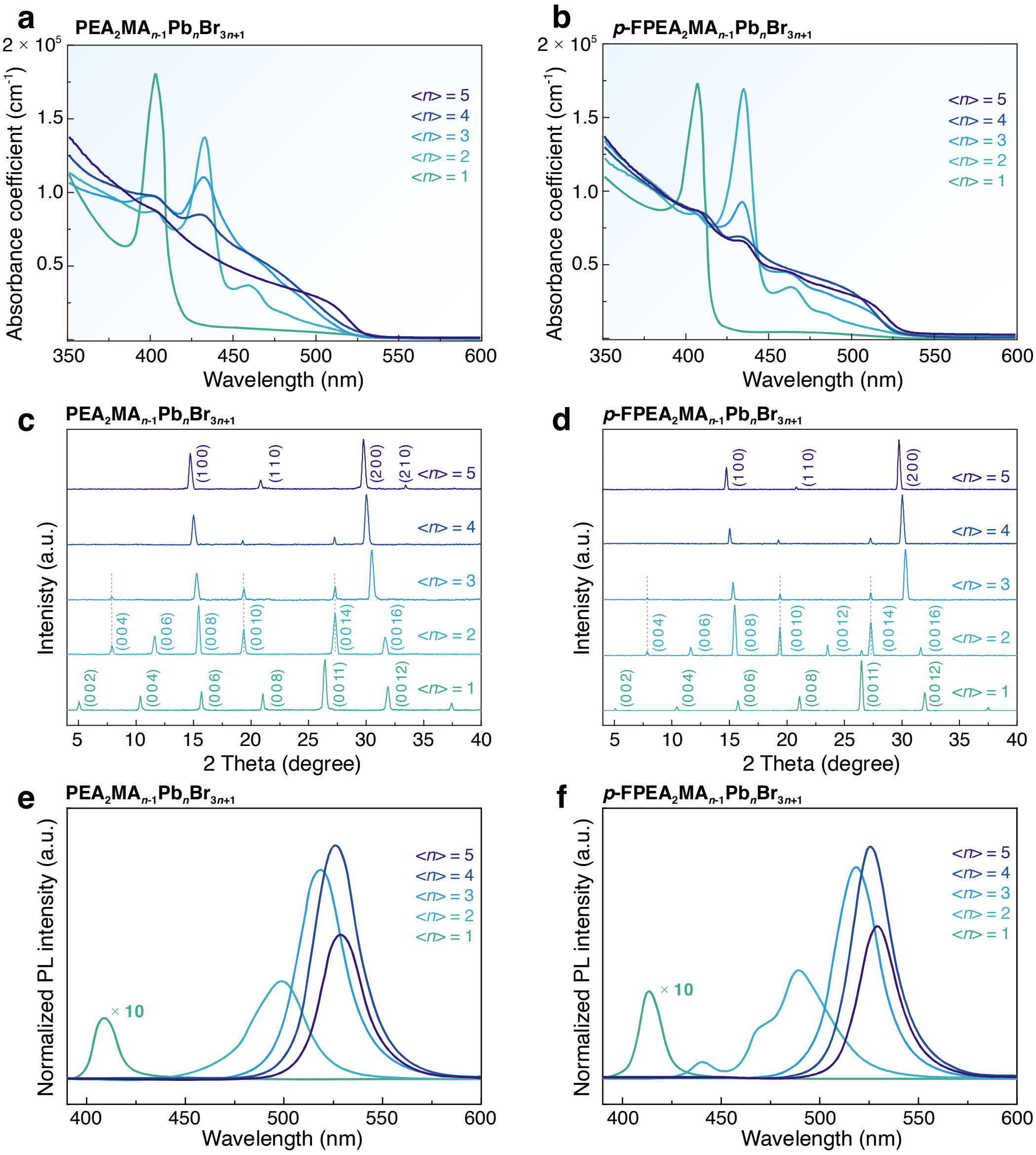

Supplementary Figure 4 | Optical and XRD characterization of quasi-2D perovskite films. (a, b) UV-vis absorption spectra, **(c, d)** XRD patterns and **(e, f)** steady-state PL spectra of **(a, c, e)** $PEA_2MA_{n-1}Pb_nBr_{3n+1}$ and **(b, d, f)** *p*-$FPEA_2MA_{n-1}Pb_nBr_{3n+1}$ perovskite films with different <*n*>-values ($1 \leq n \leq 5$). We adopt that '*n*' stands for the species with a fixed composition but '<*n*>' represents a quasi-2D perovskite domain. The absorption spectra are calculated through Supplementary equation **13**. Transmission and reflection spectra for $PEA_2MA_{n-1}Pb_nBr_{3n+1}$ and *p*-$FPEA_2MA_{n-1}Pb_nBr_{3n+1}$ quasi-2D perovskite films with different <*n*>-values are shown in Supplementary Figure 5. The (0 0 k) diffraction peaks derived from *n* = 1 and 2 species can be observed in <*n*> = 1, 2 and 3

perovskites, respectively (Supplementary Figure 4c, d), demonstrating the formation of layered structure. With the <*n*>-values increasing, the diffraction peaks of high *n* space appear, which confirms the mixed-phase within the quasi-2D perovskite films.

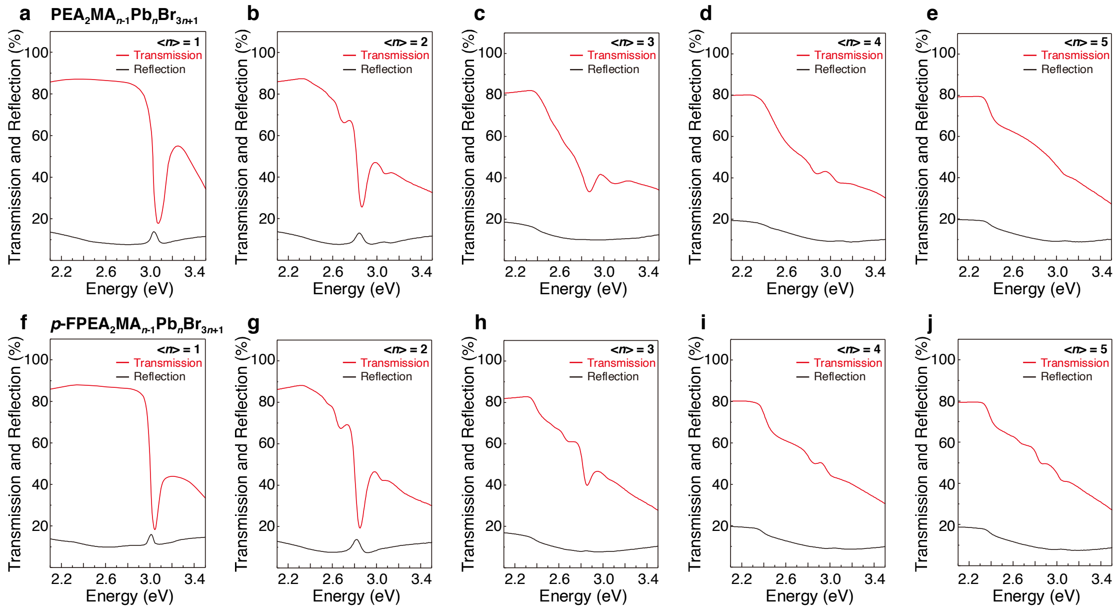

Supplementary Figure 5 | Transmission and reflection spectra. Transmission and reflection spectra for PEA$_2$MA$_{n-1}$Pb$_n$Br$_{3n+1}$ and *p*-FPEA$_2$MA$_{n-1}$Pb$_n$Br$_{3n+1}$ quasi-2D perovskite films with different <*n*> values ($1 \leq n \leq 5$).

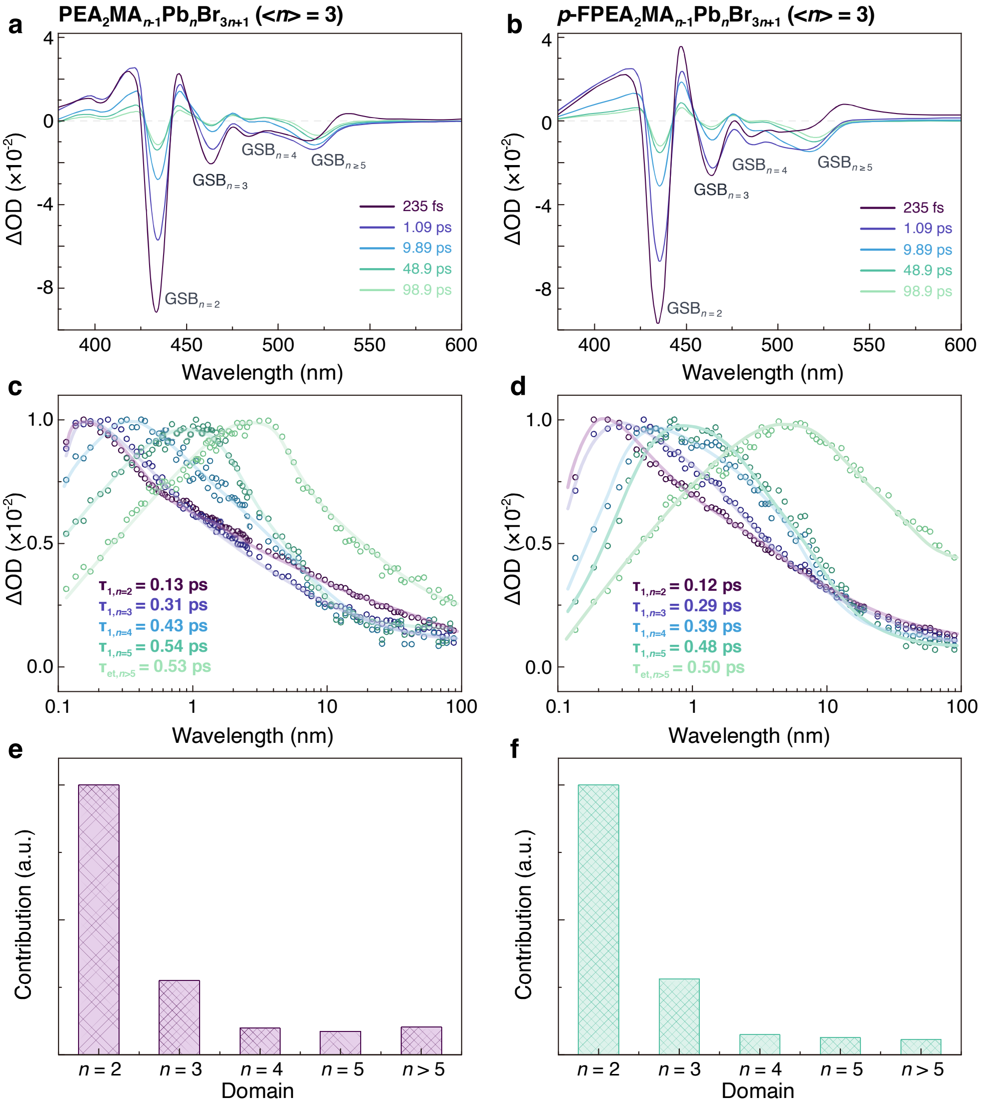

Supplementary Figure 6 | TA measurements for quasi-2D perovskites. (**a, b**) TA spectra at selected timescales; (**c, d**) TA spectra at different wavelength as a function of delay time; and (**e, f**) relative presence of different *n* domain according to the amplitude of GSBs in TA spectra at around 200 fs for (**a, c, e**) $PEA_2MA_{n-1}Pb_nBr_{3n+1}$ (<*n*> = 3) and (**b, d, f**) *p*-$FPEA_2MA_{n-1}Pb_nBr_{3n+1}$ (<*n*> = 3) films, respectively. The detailed formation time constant (τ_{et}) and first-order decay (τ_1) of each GSBs are provided in Supplementary Table 3. We qualitatively evaluated the relative presence of different *n* domains according to the amplitude of GSBs in TA spectra[5]. As shown, $PEA_2MA_{n-1}Pb_nBr_{3n+1}$ (<*n*> = 3) and *p*-$FPEA_2MA_{n-1}Pb_nBr_{3n+1}$ (<*n*> = 3) perovskite films display an almost similar domain distribution (Supplementary Figure 6e, f). Furthermore, we also extracted the energy transfer kinetics for each perovskite films

(Supplementary Figure 6c, d and Supplementary Table 3). We found that the whole energy transfer kinetics for both perovskite films occurred within sub-ps, which was significantly faster than the Auger recombination process (sub-ns)[6]. Thus, we confirm that the reduction of E_b enabled by introducing high-polarizable organic cation is the main reason for the suppressed Auger recombination, rather than the difference domain distribution or energy transfer kinetics. Similar phenomena can also be observed in quasi-2D perovskite films with $<n>$ = 4 and 5 (Supplementary Figures 7 and 8).

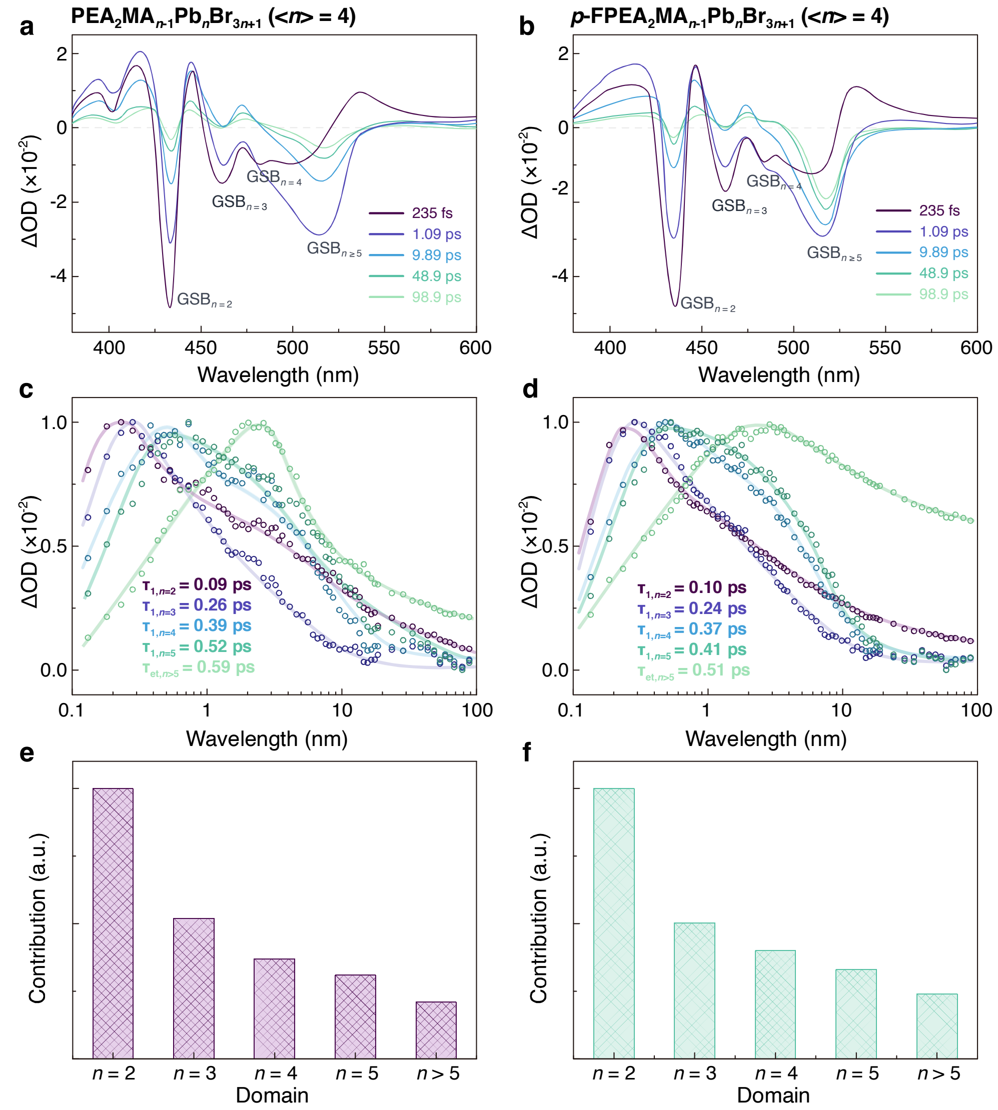

Supplementary Figure 7 | TA measurements for quasi-2D perovskites. (a, b) TA spectra at selected timescales; **(c, d)** TA spectra at different wavelength as a function of delay time; and **(e, f)** relative presence of different *n* domain according to the amplitude of GSBs in TA spectra at around 200 fs for **(a, c, e)** $PEA_2MA_{n-1}Pb_nBr_{3n+1}$ (<*n*> = 4) and **(b, d, f)** *p*-$FPEA_2MA_{n-1}Pb_nBr_{3n+1}$ (<*n*> = 4) films, respectively. The detailed formation time constant (τ_{et}) and first-order decay (τ_1) of each GSBs are provided in Supplementary Table 3.

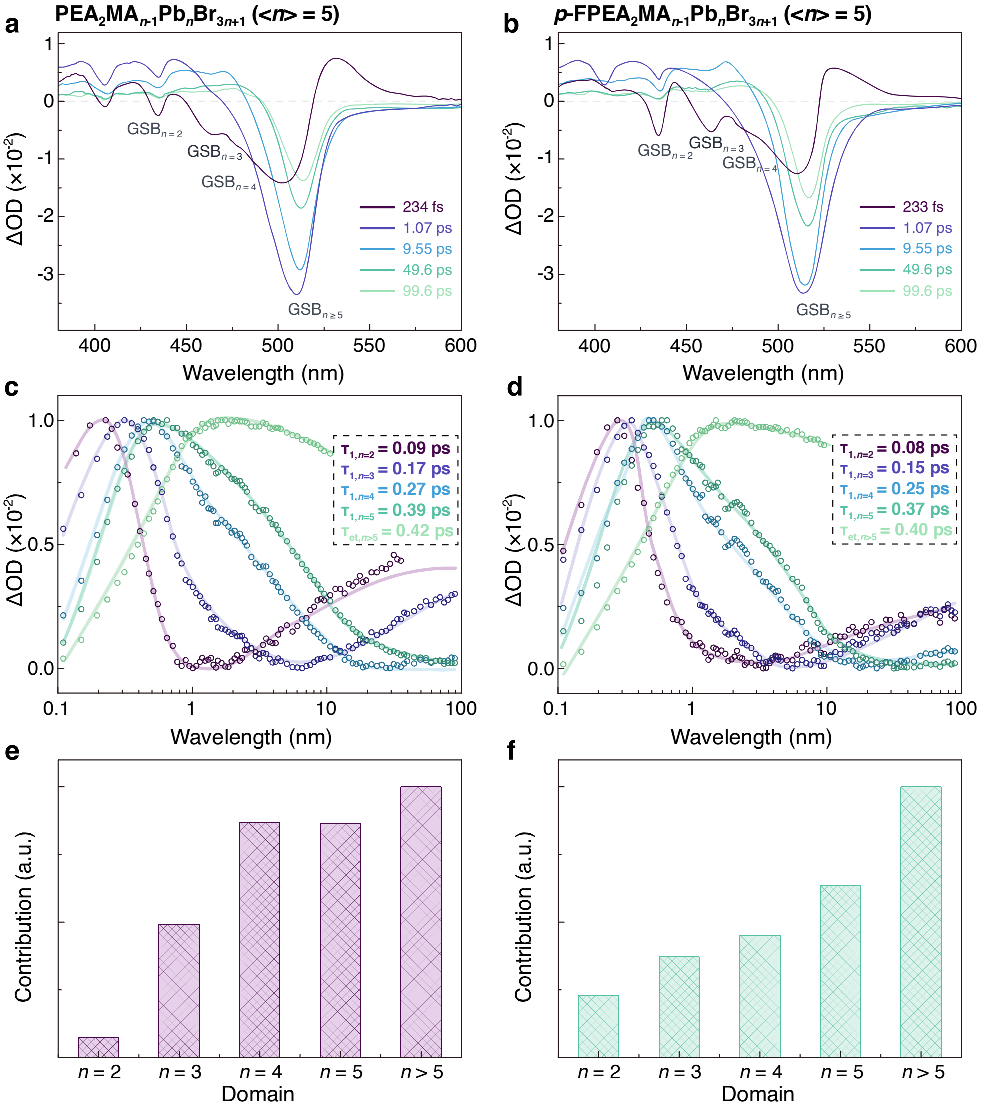

Supplementary Figure 8 | TA measurements for quasi-2D perovskites. (a, b) TA spectra at selected timescales; **(c, d)** TA spectra at different wavelength as a function of delay time; and **(e, f)** relative presence of different *n* domain according to the amplitude of GSBs in TA spectra at around 200 fs for **(a, c, e)** $PEA_2MA_{n-1}Pb_nBr_{3n+1}$ (<*n*> = 5) and **(b, d, f)** *p*-$FPEA_2MA_{n-1}Pb_nBr_{3n+1}$ (<*n*> = 5) films. The detailed formation time constant (τ_{et}) and first-order decay (τ_1) of the each GSBs are provided in Supplementary Table 3.

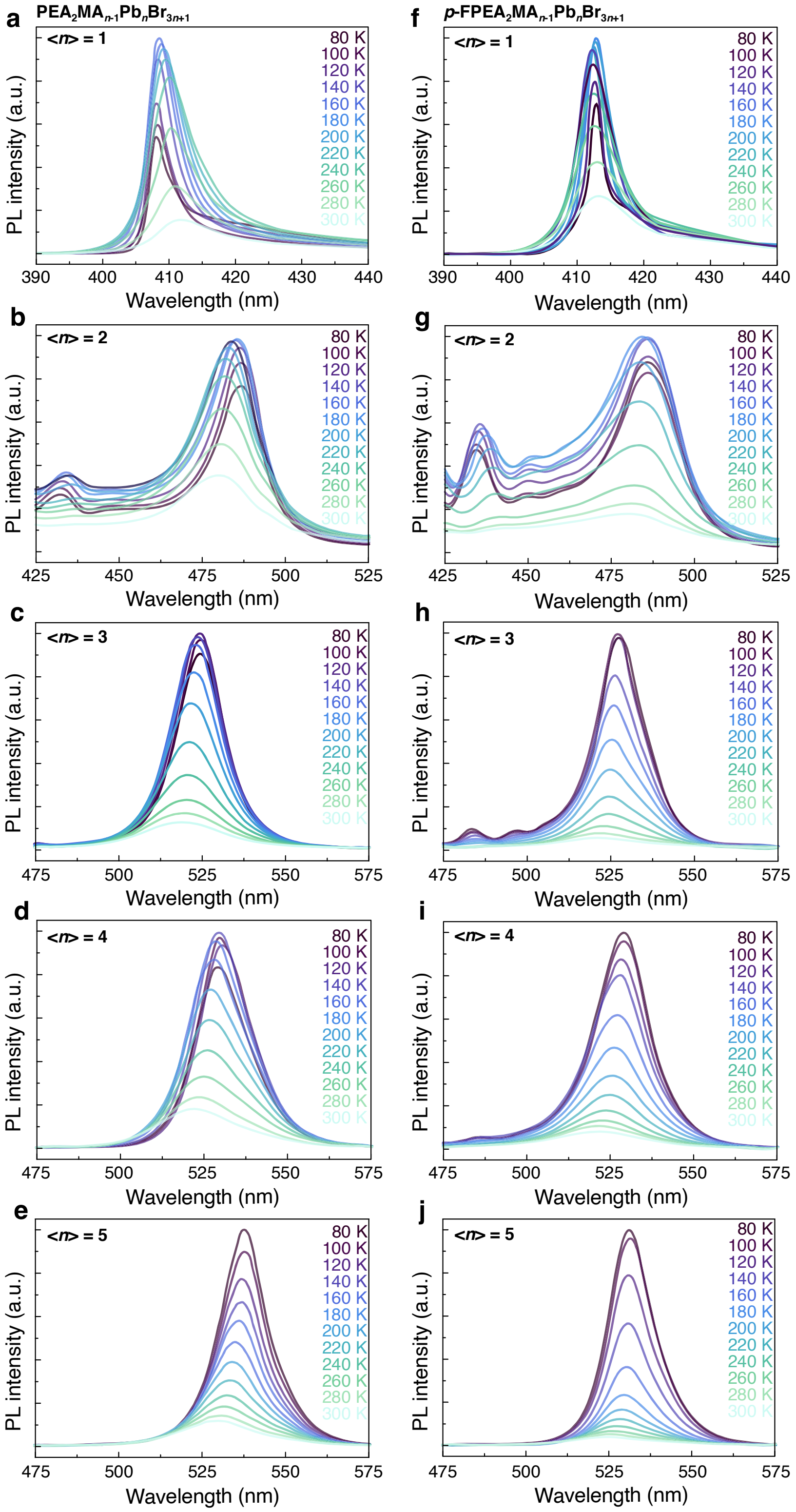

Supplementary Figure 9 | Temperature-dependent PL spectra of perovskite films with different <*n*>-values. Temperature-dependent PL spectra of **(a-e)** $PEA_2MA_{n-1}Pb_nBr_{3n+1}$ and **(f-j)** *p*-$FPEA_2MA_{n-1}Pb_nBr_{3n+1}$ perovskite films with different <*n*>-values ($1 \leq n \leq 5$).

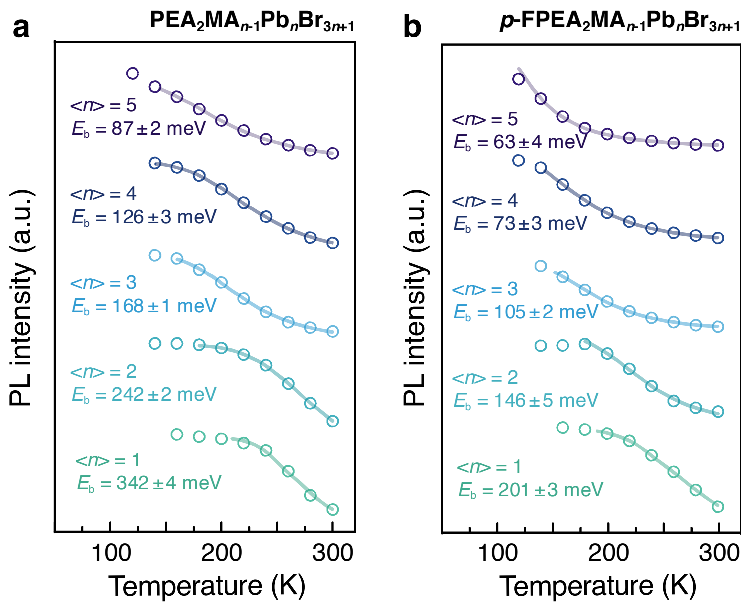

Supplementary Figure 10 | Temperature-dependent integrated PL intensity at different temperature for different perovskite films. Temperature-dependent integrating PL intensity at different temperature and corresponding fitting curves for **(a)** $PEA_2MA_{n-1}Pb_nBr_{3n+1}$ and **(b)** *p*-$FPEA_2MA_{n-1}Pb_nBr_{3n+1}$ perovskite films with different <*n*>-values ($1 \leq n \leq 5$). The exciton binding energy of each perovskite films shown in the figure is obtained through fitting according to Supplementary equation **2**.

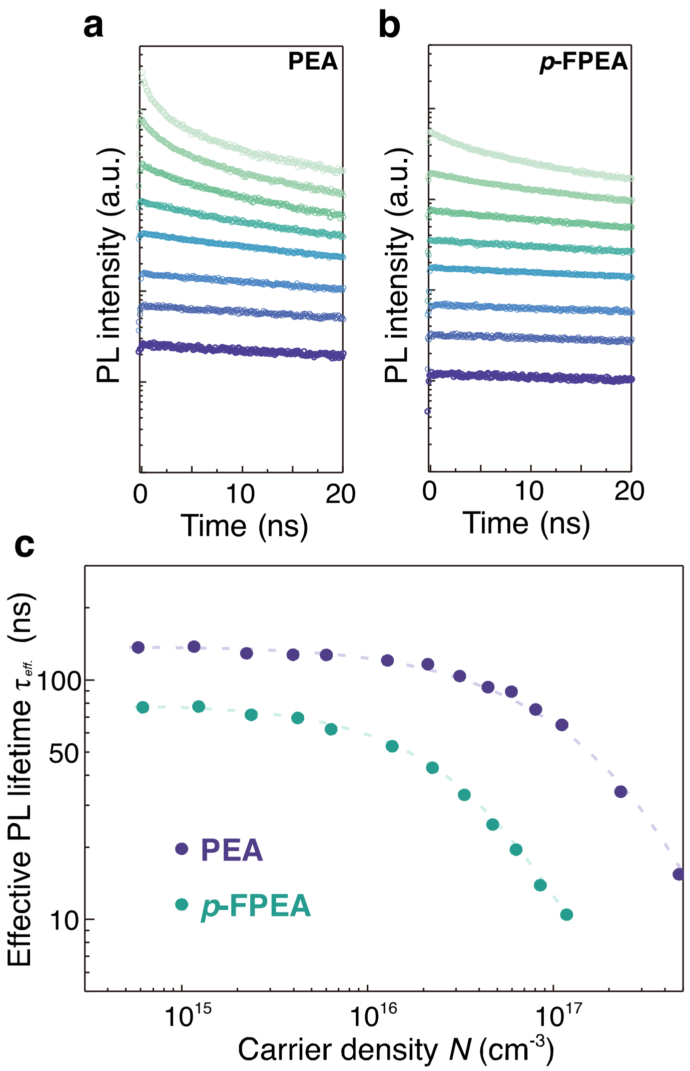

Supplementary Figure 11 | Time-resolved photoluminescence (TRPL) for perovskite films. TRPL for **(a)** PEA and **(b)** *p*-FPEA perovskite films under various excitation intensities (from 10 nJ cm^{-2} to 2.3 μJ cm^{-2}). The PL intensities at the initial time (I_{PL0}) under various excitation intensities are shown in Fig. 2a. **(c)** Effective lifetimes ($\tau_{eff.}$) as a function of carrier density for PEA and *p*-FPEA perovskite films.

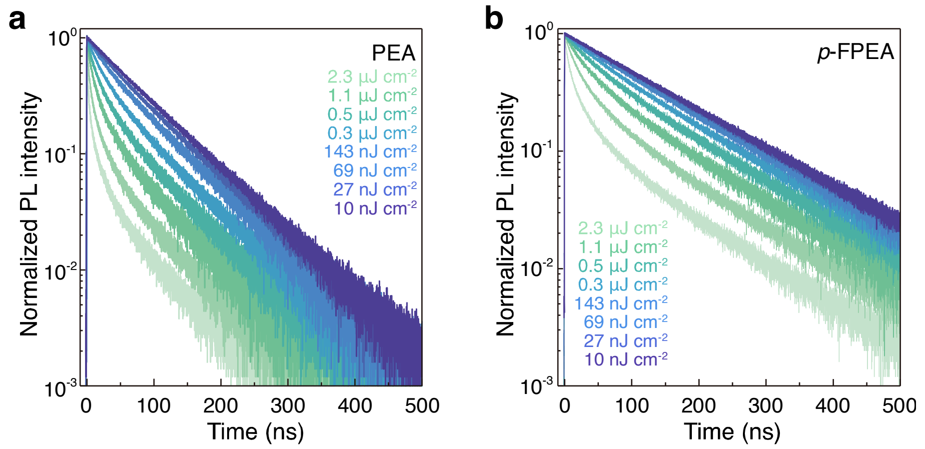

Supplementary Figure 12 | Time-resolved photoluminescence (TRPL) decay transient for perovskite films. TRPL for **(a)** PEA and **(b)** *p*-FPEA perovskite films under various excitation intensities (from 10 nJ cm^{-2} to 2.3 µJ cm^{-2}) with normalized PL intensity.

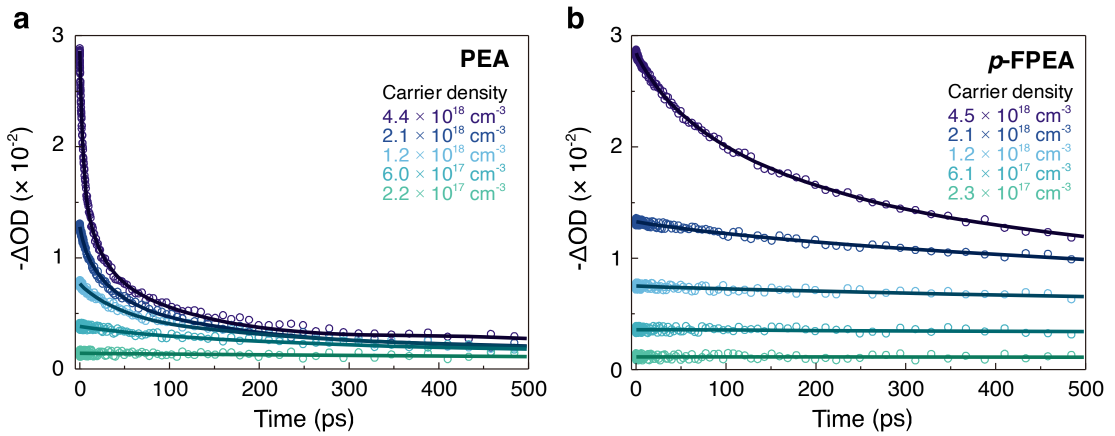

Supplementary Figure 13 | TA spectra with different pumping fluences. The TA bleaching kinetics at emission wavelength measured under various pump fluences for **(a)** PEA and **(b)** *p*-FPEA perovskite films. According to the well-established method[7], the second order (k_2) and third order (k_3) can be fitted through global fitting, and the results have been displayed in Table 1.

The global fits to transient absorption dynamics are followed the method[7]. The recombination dynamics of the charge-carrier density $N(t)$ can be described in the form of:

$$\frac{dN(t)}{dt} = -k_1 N - k_2 N^2 - k_3 N^3 \tag{3}$$

Generally, the observed quantity of photo-generated transmission change $x(t) = \frac{\Delta T}{T}(t)$ shows a linear relationship related to the charge-carrier, which can be described by the following equation:

$$N(t) = \varphi C x(t) \tag{4}$$

where φ is the photo-to-carrier conversion ratio, $C = \frac{N_0}{x(0)}$ is the proportionality factor between the initial transmission change $x(0)$ and the initially absorbed photon density N_0, which as described in Supplementary Method **1**.

Combining the Supplementary equations **3** and **4**, we obtain the following equation:

$$\frac{dN(t)}{dt} = -k_1 x - C\varphi k_2 x^2 - C^2\varphi^2 k_3 x^3 \tag{5}$$

Through a global fitting of the transient absorption dynamics in differing pump fluences by use of Supplementary equation **5**, we can extract the values of $\varphi^2 k_3$ and φk_2 according with k_1 which has been extracted by mono-exponential fitting from TRPL in a low pump density (Fig. 2e). Notably, due to the constant of φ is the ratio of photo-generated carrier density to the absorbed photon density, and its detailed value is unknown ($0 \leq \varphi \leq 1$). Thus, the k_3 and k_2 discussed in this study represent the lower limits of the actual decay constants of ($\varphi^2 k_3$ and φk_2).

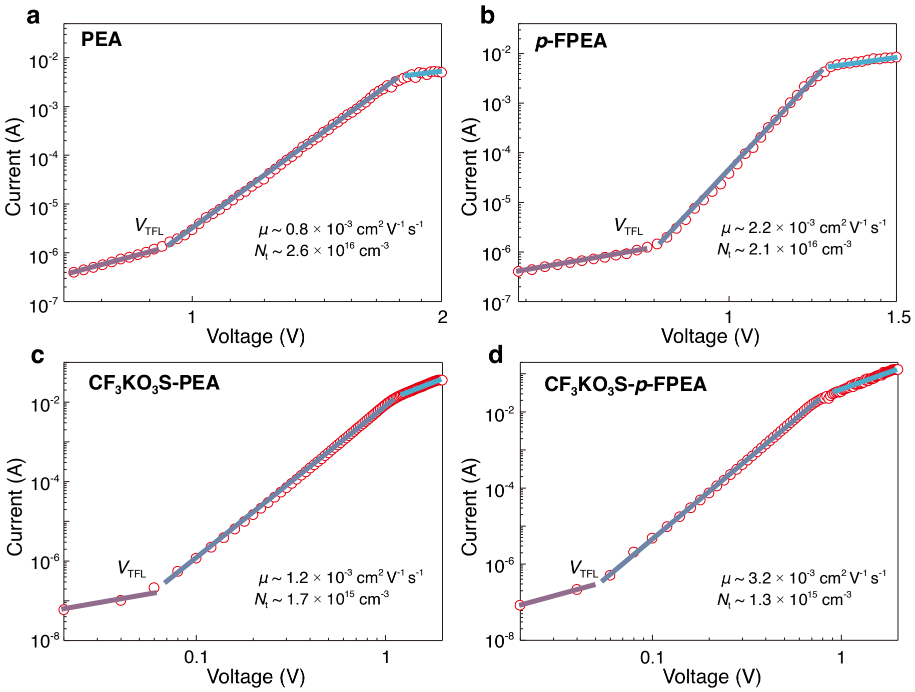

Supplementary Figure 14 | SCLC characterizations for quasi-2D perovskite films. Current-voltage (*J-V*) response for **(a)** PEA, **(b)** *p*-FPEA, **(c)** CF_3KO_3S-PEA and **(d)** CF_3KO_3S-*p*-FPEA perovskite films in space charge limited current (SCLC) measurements. The defect density and hole mobility of each films have been labeled in each figure.

Defect density of the perovskite film is carried out by the SCLC model, using a device structure of ITO/PEDOT:PSS/perovskite/MoO_3/Au[8,9]. The defect density (N_t) is calculated by the equation of [8]:

$$N_t = \frac{2\varepsilon_0\varepsilon_r V_{\mathrm{TFL}}}{ql^2} \tag{6}$$

where V_{TFL} is the trap-filled limit (TFL) voltage, q is the elementary charge, l is the film thickness, ε_r is the dielectric constant and ε_0 is the vacuum permittivity.

The hole mobility (μ) can be then extracted from the child region according to the equation of [8]:

$$I = \frac{9\varepsilon_0\varepsilon_r\mu S V^2}{8l^3} \tag{7}$$

where I is the device current, S is the electrode area and l is the film thickness.

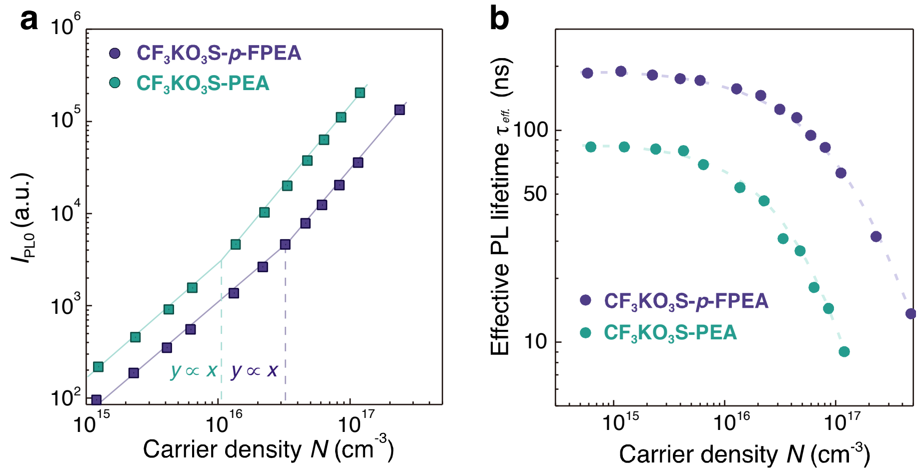

Supplementary Figure 15 | Initial time PL intensities (I_{PL0}) and effective PL lifetime at different carrier densities for different perovskite films. (a) Initial time PL intensities (I_{PL0}) as a function of carrier density and **(b)** effective PL lifetime ($\tau_{eff.}$) at different carrier densities, for CF_3KO_3S-PEA and CF_3KO_3S-*p*-FPEA films, respectively. The fitted first-order recombination rates ($k_{exciton}$ + k_{trap}) have been decreased to 1.1 × 10^7 s^{-1} and 5.3 × 10^6 s^{-1} for treated *p*-FPEA films, compared with the untreated one. In consideration of the I_{PL0} for CF_3KO_3S-PEA and CF_3KO_3S-*p*-FPEA perovskite films are almost identical; we confirm that the k_1 difference between treated and untreated films is induced by the declining of the k_{trap}.

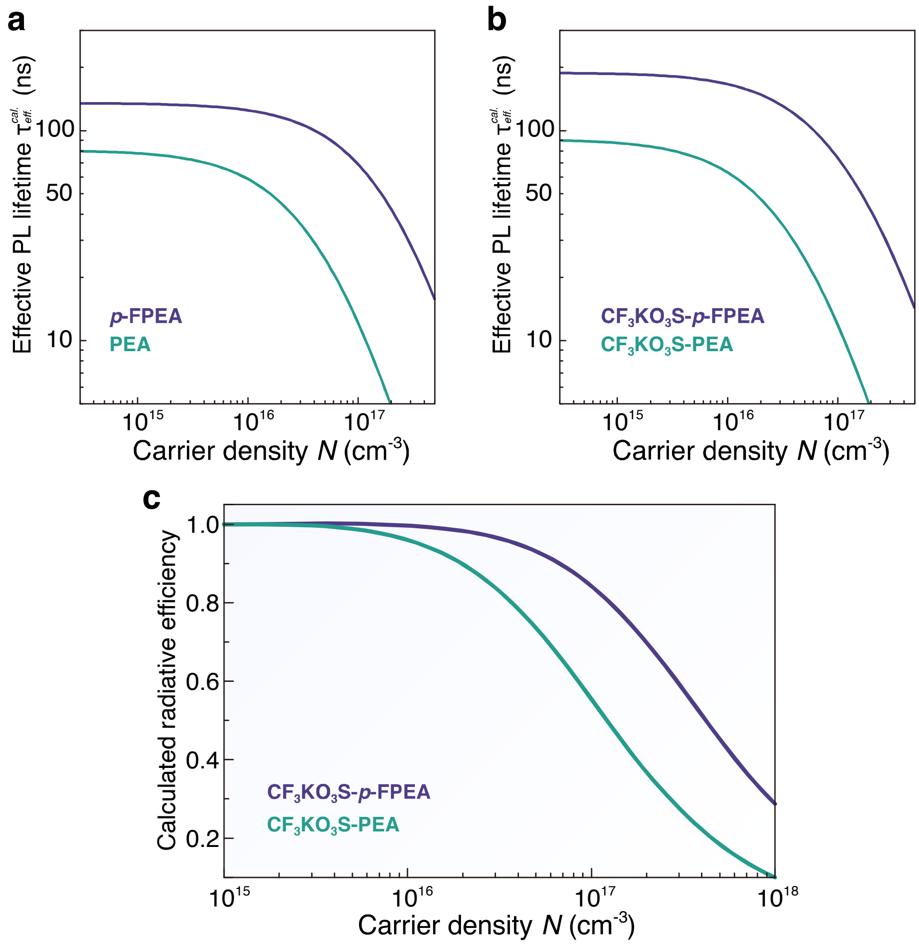

Supplementary Figure 16 | Calculated PL lifetime and luminescence quantum yield (QY) of the perovskite films. Calculated PL effective lifetimes ($\tau_{eff.}^{cal.}$) as a function of carrier density for **(a)** PEA, *p*-FPEA, **(b)** CF_3KO_3S-PEA and CF_3KO_3S-*p*-FPEA perovskite films. The calculated evolution of the $\tau_{eff.}^{cal.}$ are consistent well with the experimental results (Supplementary Figures 11c and 15b). **(c)** Calculated luminescence quantum yields [$\eta(N)$] as a function of carrier density for CF_3KO_3S-PEA and CF_3KO_3S-*p*-FPEA perovskite films. The calculated evolution of the $\eta(N)$ is consistent with the experimental results (Fig. 3c).

The high-order recombination rates (k_2 and k_3) are resolved through a global fitting procedure on fs-TA kinetics[7]. Meanwhile, the calculated evolution of the $\tau_{eff.}^{cal.}$ is consistent well with the experimental results from the TRPL kinetics, especially at high carrier densities. This result undoubtedly demonstrates the consistency between experimental results and carrier dynamic simulations. Furthermore, the kinetics is also used to simulate the luminescence quantum yield for perovskite films. As expected,

the calculated evolution of the $\eta(N)$ is also consistent with the experimental results, which reconfirm the scientificity of the photophysical model in manuscript[6].

The calculated $\tau_{eff.}^{cal.}$ and calculated $\eta(N)$ simulated from the recombination rate constants according to the Supplementary equations **8** and **9**.

The calculated effective lifetime $\tau_{eff.}^{cal.}$ can be obtained from the following equation[6]:

$$\tau_{eff.}^{cal.} = \frac{1}{k_1 + k_2 N_0/2 + k_3 {N_0}^2/3} \tag{8}$$

The calculated luminescence quantum yield of the perovskite films can be calculated according to the following equation[6]:

$$\eta(N) = \frac{k_{exciton} + Nk_2}{k_{exciton} + Nk_2 + N^2 k_3} \tag{9}$$

where k_{exciton}, k_2 and k_3 are the exciton order, second order and Auger recombination rate constant, respectively. N is the charge-carrier density. The k_{trap} is reduced to several times or one order of magnitude lower level through passivation, and could be neglected compared with k_{exciton}. Thus, we here attribute the k_1 for CF_3KO_3S-PEA and CF_3KO_3S-*p*-FPEA perovskites mainly to the k_{exciton}.

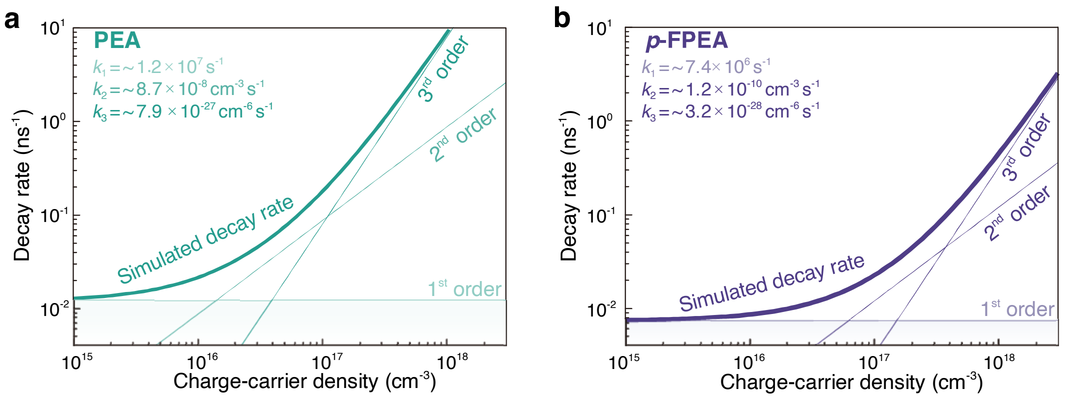

Supplementary Figure 17 | Simulated charge-carrier decay rates for perovskite films. The simulated charge-carrier decay rates of different recombination pathways and corresponding total decay rates as a function of carrier concentration for **(a)** PEA and **(b)** *p*-FPEA perovskite films.

Total charge carrier recombination rate R_{total} can be determined by the charge carrier density and the recombination rate constants (k_1, k_2 and k_3) of the individual recombination process[10]:

$$R_{total} = -\frac{1}{N}\frac{dN}{dt} = k_3N^2 + k_2N + k_1 \quad (10)$$

where R_{total}, k_1, k_2 and k_3 are plotted in the Fig. 3f and Supplementary Figure 17.

We collected each recombination rate constants of the perovskite films and then simulated their contributions to the total recombination dynamics. The total recombination dynamic is dominated by first-order recombination in the low charge carrier density range. Meanwhile, the excitons dissociate to free charge carriers at medium charge carrier density range, and bimolecular recombination pathway dominate the total recombination. With the further increased charge carrier density, Auger recombination become the fastest process. These results are consistent well with the I_{PL0} and $\tau_{eff.}$ evolution (Fig. 2a and Supplementary Figure 11c). The Auger recombination for *p*-FPEA perovskite films shifts to a higher charge carrier density range compared with that of PEA perovskite, representing the suppressed Auger

recombination derived from the reduced exciton binding energy. Similar phenomena can be also observed in CF_3KO_3S-PEA and CF_3KO_3S-*p*-FPEA perovskites.

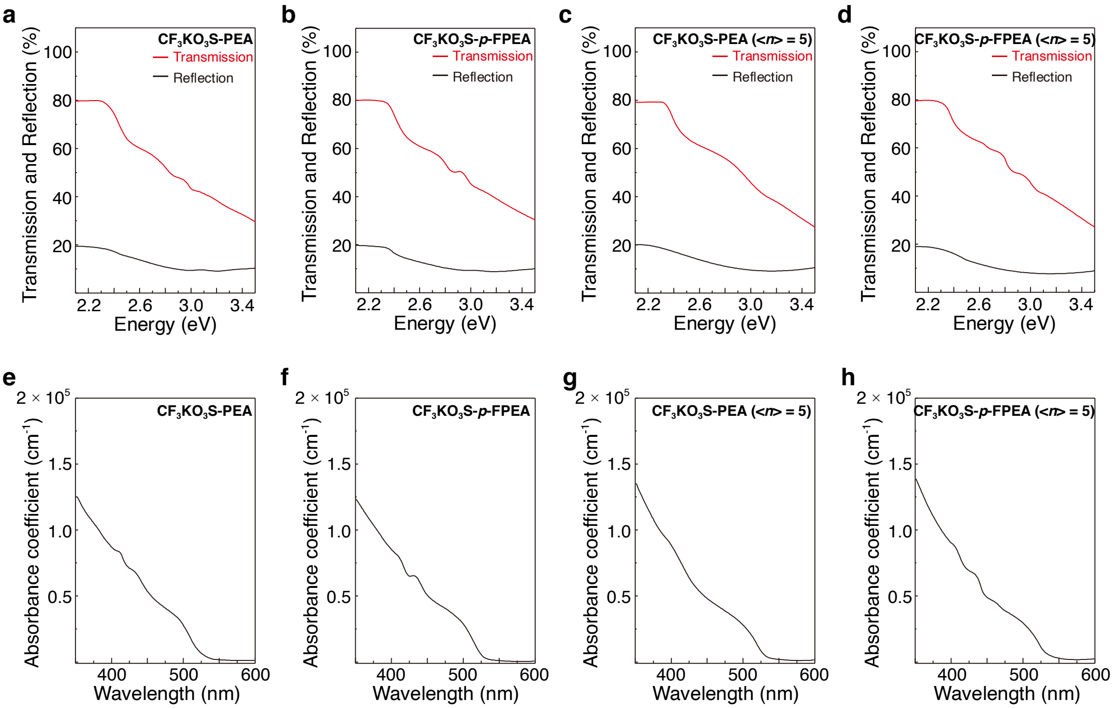

Supplementary Figure 18 | Transmission, reflection and absorption spectra for quasi-2D perovskite films. (a-d) Transmission, reflection and **(e-h)** absorption spectra for CF_3KO_3S-$PEA_2MA_{n-1}Pb_nBr_{3n+1}$ and CF_3KO_3S-*p*-$FPEA_2MA_{n-1}Pb_nBr_{3n+1}$ quasi-2D perovskite films with different <*n*>-values (<*n*> = 4 and 5).

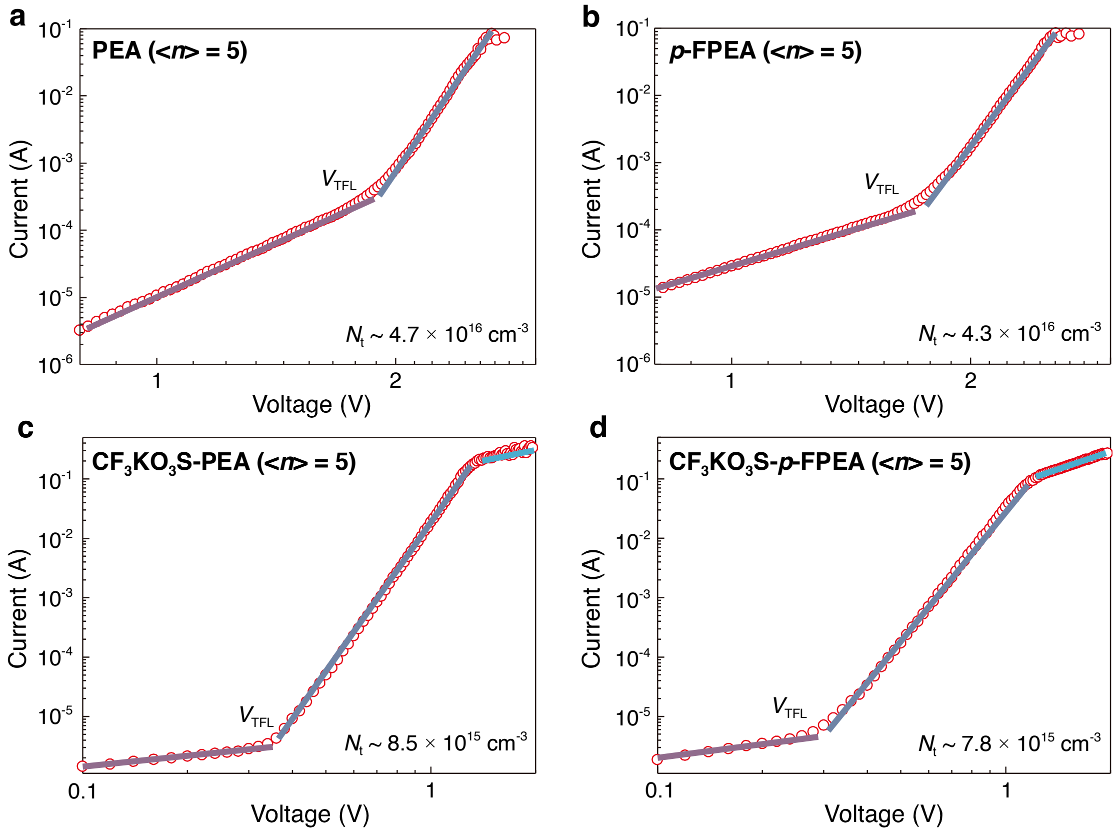

Supplementary Figure 19 | SCLC characterizations for quasi-2D perovskite films. Current-voltage (*J*-*V*) response for $PEA_2MA_{n-1}Pb_nBr_{3n+1}$ and *p*-$FPEA_2MA_{n-1}Pb_nBr_{3n+1}$ PEA (**a, b**) without and **(c, d)** with CF_3KO_3S treated perovskite films in space charge limited current (SCLC) measurements. The defect density of each film has been labeled in each figure. Defect density of the perovskite film is carried out by Supplementary equation **6**.

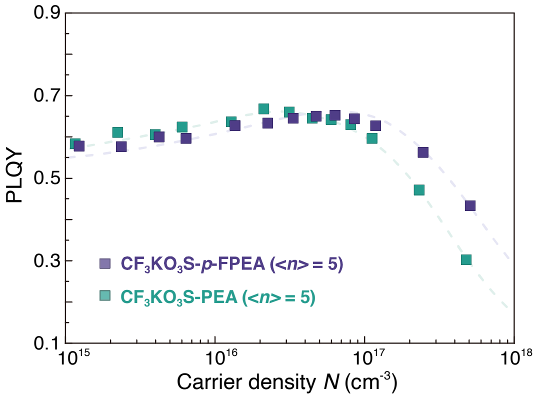

Supplementary Figure 20 | PLQYs of quasi-2D perovskite films. Film's PLQY as a function of carrier density for $CF_3KO_3S\text{-}PEA_2MA_{n-1}Pb_nBr_{3n+1}$ (<*n*> = 5) and CF_3KO_3S-*p*-$FPEA_2MA_{n-1}Pb_nBr_{3n+1}$ (<*n*> = 5) quasi-2D films, respectively.

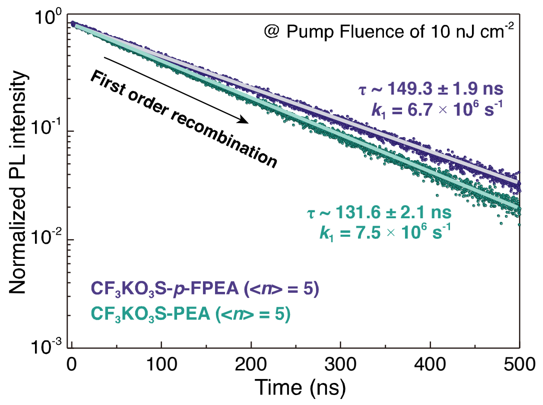

Supplementary Figure 21 | Time-resolved photoluminescence (TRPL) for quasi-2D perovskite films. TRPL for CF_3KO_3S-$PEA_2MA_{n-1}Pb_nBr_{3n+1}$ (<*n*> = 5) and CF_3KO_3S-*p*-$FPEA_2MA_{n-1}Pb_nBr_{3n+1}$ (<*n*> = 5) perovskite films under low excitation intensity of around 10 nJ cm^{-2}. Under low fluences, the PL decays follow a single-exponential behavior. The fitted first-order recombination constants, k_1, are determined to be 7.5×10^6 s^{-1} and 6.7×10^6 s^{-1} for CF_3KO_3S-$PEA_2MA_{n-1}Pb_nBr_{3n+1}$ (<*n*> = 5) and CF_3KO_3S-*p*-$FPEA_2MA_{n-1}Pb_nBr_{3n+1}$ (<*n*> = 5), respectively.

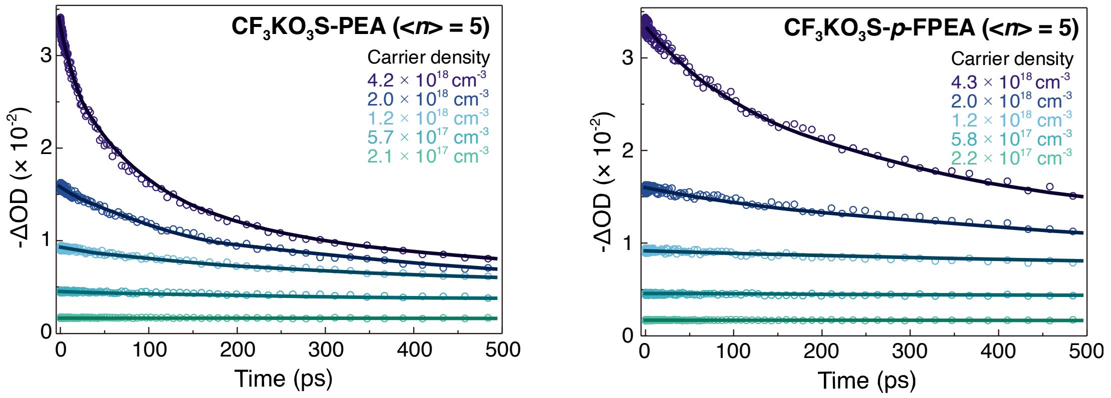

Supplementary Figure 22 | TA spectra with different pumping fluences. TA bleaching kinetics at emission wavelength measured under various pump fluences for **(a)** CF_3KO_3S-$PEA_2MA_{n-1}Pb_nBr_{3n+1}$ (<n> = 5) and **(b)** CF_3KO_3S-*p*-$FPEA_2MA_{n-1}Pb_nBr_{3n+1}$ (<n> = 5) perovskite films. According to the well-established method[3], the second order (k_2) and third order (k_3) can be fitted through global fitting (Supplementary equation **5**), and the results have been displayed in Supplementary Table 5.

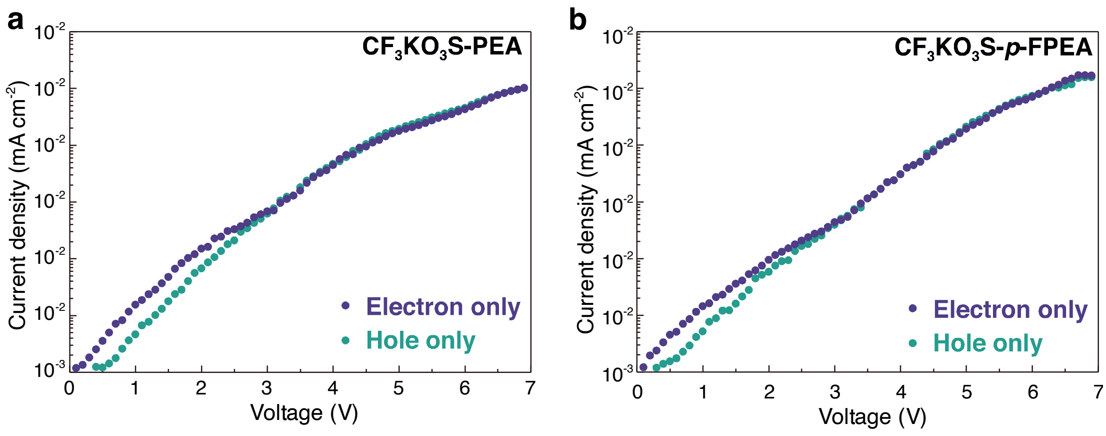

Supplementary Figure 23 | Current density-voltage (*J-V*) curves of electron-only and hole-only devices. Current density-voltage (*J-V*) curves of electron-only (ITO/ TmPyPB/PFNBr/Perovskite/PMMA/TmPyPB/Al) and hole-only (ITO/PEDOT:PSS/ PFNBr/Perovskite/PMMA/MoO_3/Au) devices for **(a)** CF_3KO_3S-PEA and **(b)** CF_3KO_3S-*p*-FPEA perovskites, respectively. The current density between these devices is basically in the same magnitude under operating voltage range, demonstrating the balanced charge injection[11].

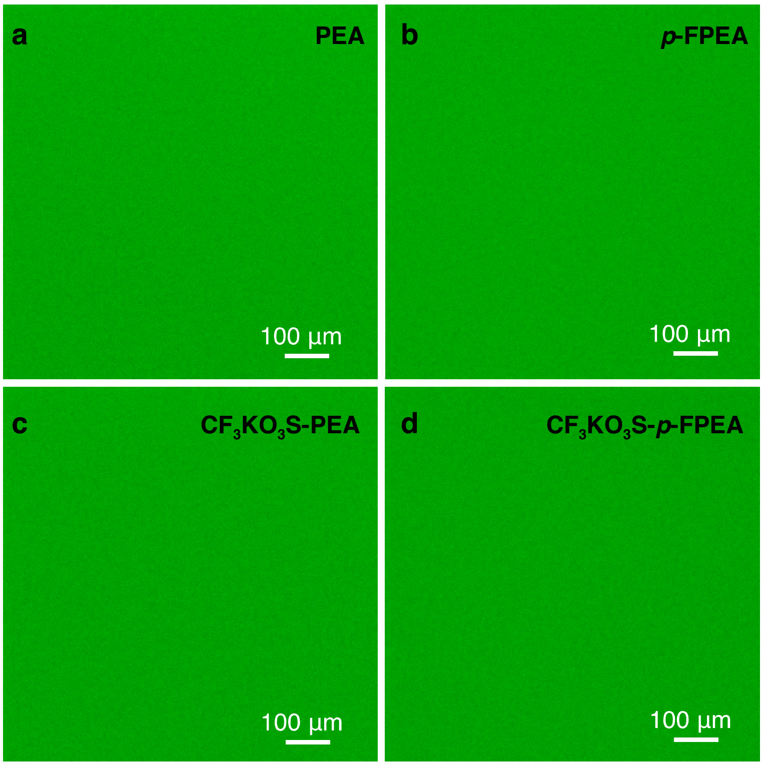

Supplementary Figure 24 | Confocal fluorescence microscopy (CFM) images of different perovskite films. CFM images of **(a)** PEA, **(b)** *p*-FPEA, **(c)** CF_3KO_3S-PEA and **(d)** CF_3KO_3S-*p*-FPEA perovskite films, respectively. The scale of all images is 0.85 mm × 0.85 mm.

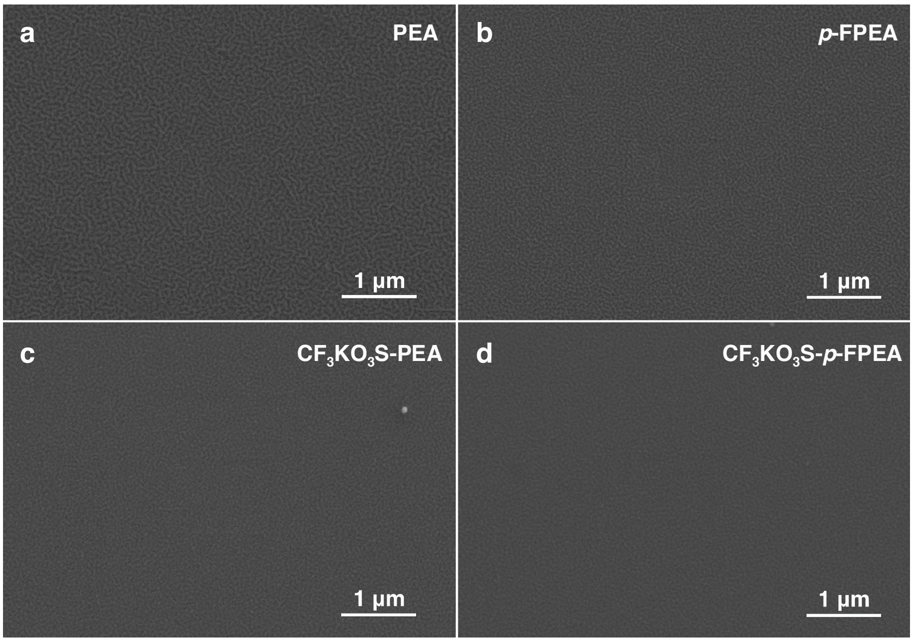

Supplementary Figure 25 | Top-view SEM images of different perovskite films. Top-view SEM images for **(a)** PEA, **(b)** *p*-FPEA, **(c)** CF_3KO_3S-PEA and **(d)** CF_3KO_3S-*p*-FPEA perovskite films coated on PEDOT:PSS layer.

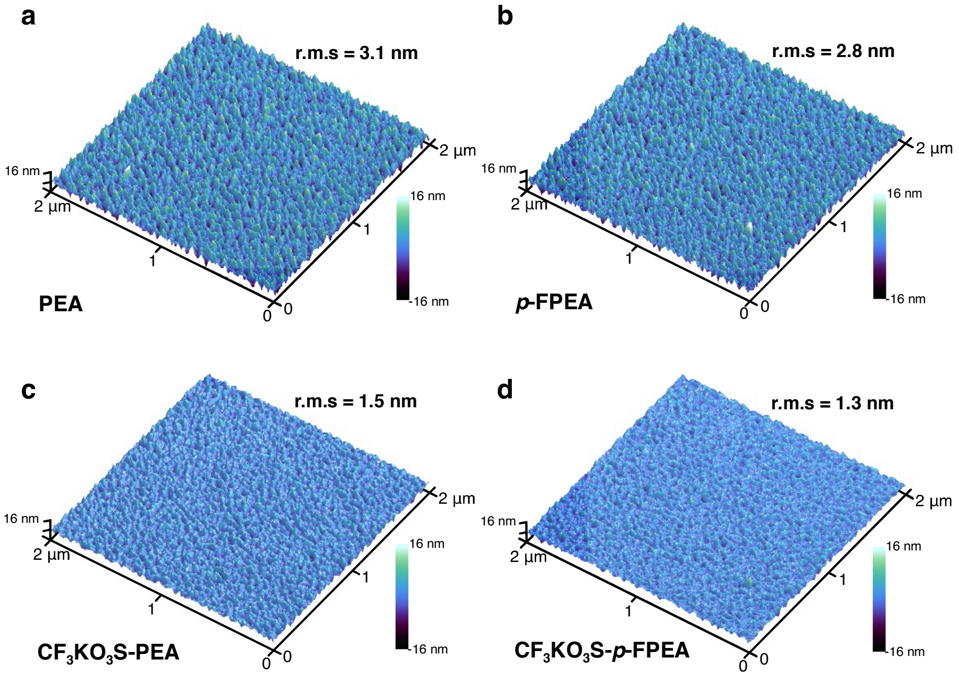

Supplementary Figure 26 | AFM images of different perovskite films. AFM images for **(a)** PEA, **(b)** *p*-FPEA, **(c)** CF_3KO_3S-PEA and **(d)** CF_3KO_3S-*p*-FPEA perovskite films coated on PEDOT:PSS layer. The r.m.s. roughness of the films are marked on the right sides of the images, respectively. The scan of all images is 2 μm × 2 μm.

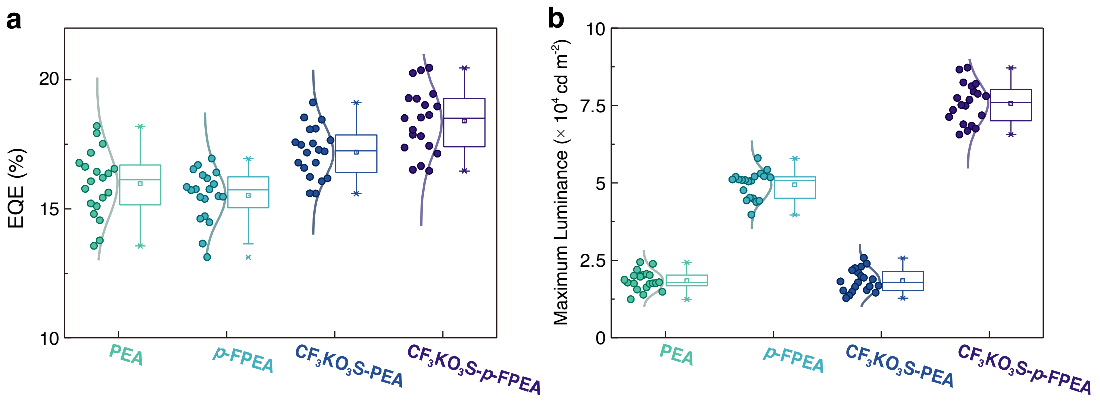

Supplementary Figure 27 | Histograms of peak EQE and maximum luminance. **(a)** EQE and **(b)** maximum luminance statistics for PEA, *p*-FPEA, CF_3KO_3S-PEA and CF_3KO_3S-*p*-FPEA PeLEDs.

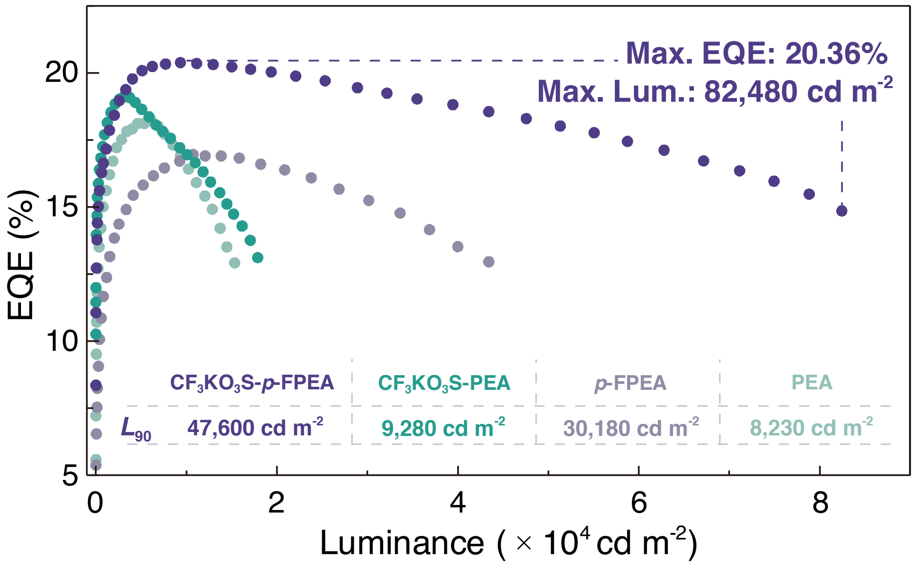

Supplementary Figure 28 | External quantum efficiency (EQE) versus luminance curves of different PeLEDs. External quantum efficiency (EQE) versus luminance curves of PeLEDs based on PEA, *p*-FPEA, CF_3KO_3S-PEA and CF_3KO_3S-*p*-FPEA perovskites. The L_{90} (defined as the luminance for which the EQE drops to 90% of its maximum value) values of corresponding PeLED devices are marked in the figure.

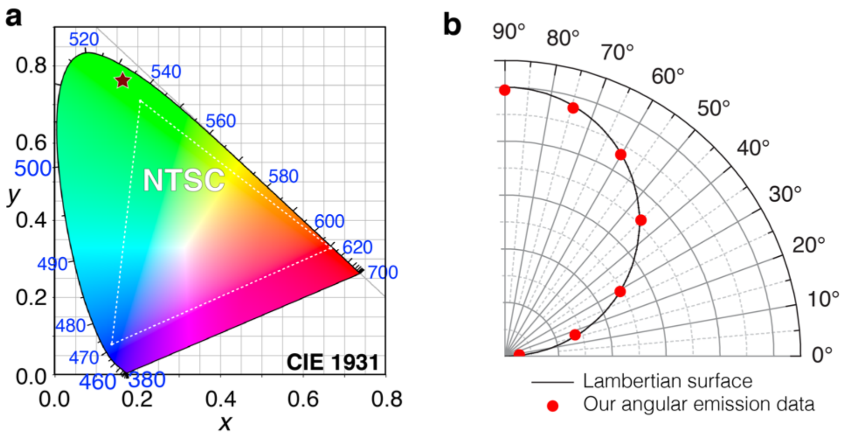

Supplementary Figure 29 | Electroluminescence (EL) properties of PeLED devices. (a) Commission Internationale de I'Echlaiage (CIE) coordinate for our PeLEDs. **(b)** Angular distribution of radiation intensity for a Lambertian emitter and our PeLEDs. The detector is deposited at a fixed distance of 200 mm from the EL device. The PeLED based on CF_3KO_3S-*p*-FPEA exhibits an excellent color purity with the CIE chromatic coordinates of (0.162, 0.758), which is close to the NTSC values. The PeLED also follows a Lambertian profile.

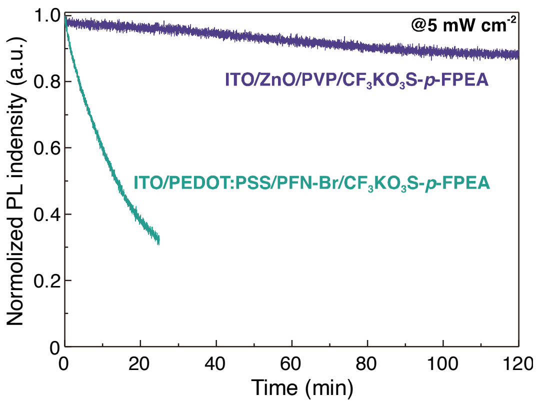

Supplementary Figure 30 | PL stability of perovskite films. PL intensity of the quasi-2D perovskite films coated on different charge transfer layers under continuous excitation of 5 mW cm^{-2}. It has been reported that the interfacial contact between the perovskite and charge transport layers is a key factor for the device's operational stability[12]. As shown, CF_3KO_3S-*p*-FPEA perovskite coated on ZnO/PVP layer remains 90% of its initial the PL intensity for even 120 min. However, PL intensity of the perovskite coated on PEDOT:PSS/PFN-Br layer displays a sharp decline. It has been demonstrated that the acidic nature of PEDOT:PSS could significantly corrode the active layers[12]. Thus, we attribute the loss of devices' efficiency to the chemical instability at the interface between perovskite and charge transport layers, rather than to perovskite degradation itself.

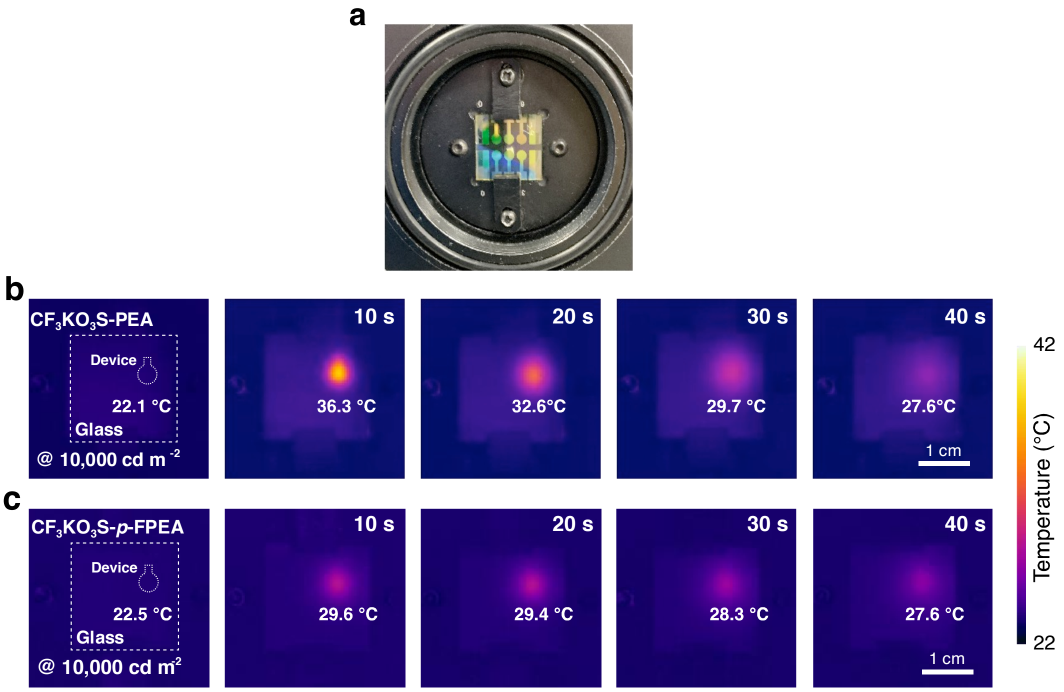

Supplementary Figure 31 | Thermal imaging measurement for PeLEDs. (a) Photograph of the test fixture and the PeLED device which for thermal imaging measurements. (**b, c**) Spatial surface temperature images of the glass substrate monitored at various time points (0, 10, 20, 30 and 40 s) for PeLEDs based on **(b)** CF_3KO_3S-PEA and **(c)** CF_3KO_3S-*p*-FPEA at a high initial luminance of 10,000 cd m^{-2}.

The CF_3KO_3S-PEA device exhibits an extremely high temperature of 36.3 °C only after operation for 10 s (Supplementary Figure 31b). Non-radiative Auger recombination can induce excessive heat generation, and then increase the local temperature of the device, thus we correlate the high temperature with the serious Auger recombination in perovskite layers, especially operating under such high current-injection. In addition, after reaching the maximum point, the temperature of the CF_3KO_3S-PEA device shows a sharp reduction (Supplementary Figures 31b and 32c), indicating an irreversible degradation on device structure. This result is consistent with our prior T_{50} measurement (Fig. 4e), discovering the main reason for inferior operational stability, i.e., Auger recombination. By contrast, the surface temperature of the CF_3KO_3S-*p*-FPEA device only increased to 29.6 °C after operation

for 10 s, and reached thermal equilibrium throughout the measurement period of 40 s (Supplementary Figures 31c and 32c). This is consistent with our observation that the CF_3KO_3S-*p*-FPEA device possesses a more stable operation and a longer half-lifetime compared to PEA^+ analogue.

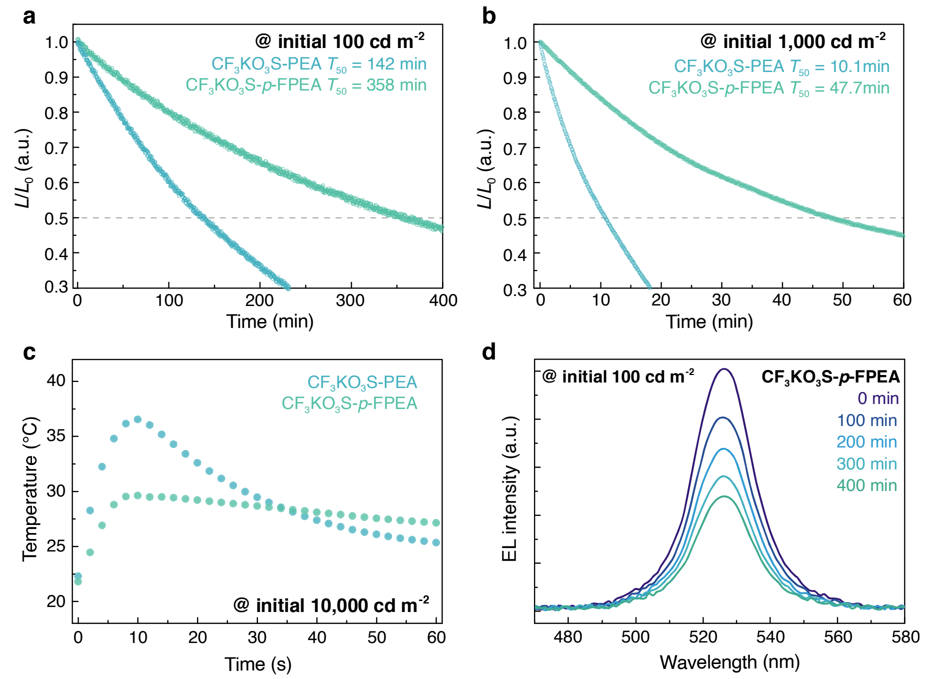

Supplementary Figure 32 | Operational stability measurements for PeLED devices. (**a, b**) Lifetime measurement (T_{50}) of the corresponding PeLED devices with an initial luminance of (**a**) 100 cd m^{-2} and (**b**) 1,000 cd m^{-2}. (**c**) The detailed spatial surface temperature of the devices is extracted from thermal imaging measurements (Supplementary Figure 31b, c) with an initial luminance of 10,000 cd m^{-2}. (**d**) EL spectrum for PeLED devices based on CF_3KO_3S-*p*-FPEA operating with various exposure time at an initial luminance of 100 cd cm^{-2}. The EL spectra of the PeLED devices are almost identical during the test, demonstrating excellent spectral stability.

Supplementary Table 1 | Perovskite precursor solutions for different compositions. The perovskite precursor solutions of $PEA_2MA_{n-1}Pb_nBr_{3n+1}$ and *p*-$FPEA_2MA_{n-1}Pb_nBr_{3n+1}$. The treated perovskite films (CF_3KO_3S-PEA and CF_3KO_3S-*p*-FPEA) can be obtained by further introducing a judicious amount of CF_3KO_3S into the precursor solutions.

Composition	PEABr/*p*-FPEABr (mL^{-1})	MABr (mL^{-1})	$PbBr_2$ (mL^{-1})	MAAc (mL^{-1})	CF_3KO_3S (mL^{-1})
$<n> = 1$	1 mmol	-	0.5 mmol	50 µL	-
$<n> = 2$	0.5 mmol	0.25 mmol	0.5 mmol	50 µL	-
$<n> = 3$	0.33 mmol	0.33 mmol	0.5 mmol	50 µL	-
$<n> = 4$	0.25 mmol	0.375 mmol	0.5 mmol	50 µL	-
$<n> = 5$	0.2 mmol	0.4 mmol	0.5 mmol	50 µL	-
CF_3KO_3S-PEA/ CF_3KO_3S-*p*-FPEA	0.25 mmol	0.375 mmol	0.5 mmol	50 µL	0.025 mmol

Supplementary Table 2 | Crystallographic data for *p*-FPEA$_2$PbBr$_4$.

	p-FPEA$_2$PbBr$_4$
Empirical Formula	$F_2C_{16}H_{22}N_2PbBr_4$
Formula Weight, g mol^{-1}	807.18
Temperature, K	296.15
Crystal System	Monoclinic
Space group	*C2/c*
a, Å	33.478(7)
b, Å	8.1466(16)
c, Å	8.4104(17)
α, °	90
β, °	96.137(2)
γ, °	90
Volume, Å^3	2280.7(8)
Z	4
Density (calculated), g cm^{-3}	2.351
Absorption coefficient, mm^{-1}	14.424
F(000)	1488.0
Crystal size, mm^3	0.2 × 0.1 × 0.1
θ range, °	2.45 to 27.34
Index ranges	$-39 \leq h \leq 39$<br>$-9 \leq k \leq 9$<br>$-10 \leq l \leq 10$
Reflections collected / unique	10071 / 1968
Completeness to θ_{max}	0.982
Max. and min. transmission	0.404, 0.746
Data / restraints / parameters	1968 / 0 / 116
Goodness-of-fit in F^2	1.076
Final R indices [I>2sigma(I)]	R1 = 0.0238<br>wR2 = 0.0539
R indices (all data)^a	R1 = 0.0298<br>wR2 = 0.0516
Largest diff. peak and hole, e Å^{-3}	0.597 and -1.389

^{a}R1 = $\Sigma||Fo|-|Fc||/\Sigma|Fo|$, wR = $\{\Sigma[w(|Fo|^2 - |Fc|^2)^2]/ \Sigma[w(|Fo|^4)]\}^{1/2}$

Supplementary Table 3 | The corresponding formation time constant (τ_{et}) and first-order decay (τ_1) of the each GSBs shown in Supplementary Figures 6-8. The kinetics are fitted by a multiple-exponential function, $\Delta A(t) = a_1\exp(-t/\tau_1) + a_2\exp(-t/\tau_2) + a_3\exp(-t/\tau_3) - c_1\exp(-t/\tau_{et})$, where a_1, a_2, a_3 and c_1 are amplitudes; τ_1, τ_2 and τ_3 are decay time constants and τ_{et} is formation time constant. Due to the complex slow decay process of τ_2 and τ_3, including energy slow transfer process, trap-assisted recombination etc., only τ_1 and τ_{et} are listed in the Table.

Composition	τ	$GSB_{n=2}$ (~435 nm)	$GSB_{n=3}$ (~462 nm)	$GSB_{n=4}$ (~483 nm)	$GSB_{n=5}$ (~490 nm)	$GSB_{n>5}$ (~516 nm)
PEA (<*n*> = 3)	τ_1	0.13 ps	0.31 ps	0.43 ps	0.54 ps	
	τ_{et}	0.08 ps	0.13 ps	0.23 ps	0.38 ps	0.53 ps
p-FPEA (<*n*> = 3)	τ_1	0.12 ps	0.29 ps	0.39 ps	0.48 ps	
	τ_{et}	0.08 ps	0.15 ps	0.21 ps	0.36 ps	0.50 ps
PEA (<*n*> = 4)	τ_1	0.09 ps	0.26 ps	0.39 ps	0.52 ps	
	τ_{et}	0.08 ps	0.12 ps	0.18 ps	0.27 ps	0.59 ps
p-FPEA (<*n*> = 4)	τ_1	0.10 ps	0.24 ps	0.37 ps	0.41 ps	
	τ_{et}	0.07 ps	0.12 ps	0.17 ps	0.25 ps	0.51 ps
PEA (<*n*> = 5)	τ_1	0.09 ps	0.17 ps	0.27 ps	0.39 ps	
	τ_{et}	0.07 ps	0.11 ps	0.15 ps	0.25 ps	0.42 ps
p-FPEA (<*n*> = 5)	τ_1	0.08 ps	0.15 ps	0.25 ps	0.37 ps	
	τ_{et}	0.07 ps	0.12 ps	0.15 ps	0.24 ps	0.40 ps

Supplementary Table 4 | Exponential fits for PL decay curves. Time constant and amplitude of perovskite films can be obtained by exponential fitting to the corresponding PL decay curves. In order to evaluate the Auger recombination kinetics of the perovskite materials, the PL decay curves are measured under the excitation intensity of 2.3 μJ cm^{-2}, and fitted by a multi-exponential function as follows:

$$A(t) = A_1 e^{-\frac{t}{\tau_1}} + A_2 e^{-\frac{t}{\tau_2}} + A_3 e^{-\frac{t}{\tau_3}} \quad (10)$$

where A_1, A_2 and A_3 are amplitude constants; t is time; and τ_1, τ_2 and τ_3 represent the decay lifetimes of carriers.

The corresponding time constants and amplitudes of PEA and *p*-FPEA perovskite films have been listed as follows:

	A_1	τ_1	A_2	τ_2	A_3	τ_3
PEA	0.073	54.2 ± 3.4 ns	0.231	9.7 ± 1.1 ns	0.652	0.8 ± 0.2 ns
p-FPEA	0.239	73.7 ± 5.4 ns	0.402	14.9 ± 1.8 ns	0.331	3.4 ± 0.2 ns

In order to obtain the lifetime and corresponding recombination rate constant (k_1) of first-order recombination pathway, the PL decay curves of perovskite films are measured under a low excitation intensity of 10 nJ cm^{-2}. The resulted PL decay curves are fitted by a mono-exponential function as following function:

$$A(t) = A_1 e^{-\frac{t}{\tau_1}} \quad (11)$$

The corresponding time constants and amplitudes of PEA, *p*-FPEA, CF_3KO_3S-PEA and CF_3KO_3S-*p*-FPEA perovskite films have been listed as follows:

	PEA	*p*-FPEA	CF_3KO_3S-PEA	CF_3KO_3S-*p*-FPEA
A_1	0.991	0.990	0.992	0.991
τ_1	80.6 ± 1.8 ns	135.2 ± 2.9 ns	90.4 ± 4.2 ns	188.6 ± 6.5 ns

Supplementary Table 5 | PLQYs (η_{PL}) under carrier density of ~ 1.2 × 10^{15} and ~ 2.3 × 10^{17} cm^{-3}, monomolecular (k_1), bimolecular (k_2), and trimolecular (k_3) recombination constant, estimated defect state density (N_t) of CF_3KO_3S-PEA (<*n*> = 5) and CF_3KO_3S-PEA (<*n*> = 5) perovskite films.

	η_{PL} †	η_{PL} ‡	k_1 (s^{-1})	k_2 (cm^3s^{-1})	k_3 (cm^6s^{-1})	N_t (cm^{-3}) §
CF_3KO_3S-PEA (<*n*> = 5)	0.58	0.46	7.5(± 0.2) × 10^6	2.1(± 0.3) × 10^{-10}	9.2(± 2.1) × 10^{-28}	8.5 × 10^{15}
CF_3KO_3S-*p*-FPEA (<*n*> = 5)	0.56	0.54	6.7(± 0.1) × 10^6	1.0(± 0.2) × 10^{-10}	2.3(± 0.9) × 10^{-28}	7.8 × 10^{15}

† Measured under the carrier density of ~ 1.2 × 10^{15} cm^{-3}.

‡ Measured under the carrier density of ~ 2.3 × 10^{17} cm^{-3}.

§ Extracted from SCLC measurements.

Supplementary Table 6 | Device parameters of our best PeLEDs with different perovskite films.

Composition	Peak EQE	Max. Lum.	L_{90}	J_{90}
PEA	18.20%	17,631 cd m^{-2}	8,230 cd m^{-2}	18.4 mA m^{-2}
p-FPEA	16.94%	52,165 cd m^{-2}	30,180 cd m^{-2}	58.3 mA m^{-2}
C_3KO_3S-PEA	19.11%	19,995 cd m^{-2}	9,280 cd m^{-2}	15.9 mA m^{-2}
C_3KO_3S-*p*-FPEA	20.36%	82,480 cd m^{-2}	47,600 cd m^{-2}	82.6 mA m^{-2}

Supplementary Table 7 | Comparison of the performance for PeLEDs with visible spectrum emission (from 400 to 760 nm; $EQE_{max} > 15\%$).

Types of Perovskites	EL	Peak EQE	Max. Lum.	L_{90}	T_{50}	Ref.
$PBABr_y(Cs_{0.7}FA_{0.3}PbBr_3)$ QWs	483 nm	9.50%	~700 cd m^{-2}	~200 cd m^{-2}	250 s @ 100 cd m^{-2}	Ref.13
$PEA_2Cs_{n-1}Pb_nBr_{3n+1}$ QWs	514 nm	15.5%	19,540 cd m^{-2}	~10,000 cd m^{-2}	60 min @ 2 mA cm^{-2}	Ref.14
$Na_2Cs_{n-1}Pb_nBr_{3n+1}$ QWs	518 nm	15.9%	11,560 cd m^{-2}	-	150 min @ 150 cd m^{-2}	Ref.15
$PEA_2(FAPbBr_3)_2PbBr_4$ QWs	532 nm	15.4%	15,765 cd m^{-2}	~12,000 cd m^{-2}	--	Ref.16
$MA_{0.1}Cs_{0.79}PbBr_3$ Bulk	518 nm	17%	35,700 cd m^{-2}	~20,000 cd m^{-2}	--	Ref.17
$CsPbBr_3$ Bulk	520 nm	16.2%	50,270 cd m^{-2}	~18,000 cd m^{-2}	6.2 min @ 10,000 cd m^{-2}	Ref.18
$CsPbBr_3$@MABr Bulk	525 nm	20.3%	14,000 cd m^{-2}	~5,000 cd m^{-2}	10.42 min @ 7,130 cd m^{-2}	Ref.19
$FA_{0.33}Cs_{0.67}Pb(I_{0.7}Br_{0.3})_3$ Bulk	694 nm	20.9%	~ 300 cd m^{-2}	~160 cd m^{-2}	14 h @ 25 cd cm^{-2}	Ref.20
$FAPbI_3$ Bulk	700 nm	15.8%	218 cd m^{-2}	-	--	Ref.21
$Cs_{0.2}FA_{0.8}PbI_{2.8}Br_{0.2}$ Bulk	752 nm	17.6%	199 W $sr^{-1}m^{-2}$	~80 W sr^{-1} m^{-2}	~180 min @ 10 mA cm^{-2}	Ref.22
$FAPbBr_3$ NPs	526 nm	16.3%	13,970 cd m^{-2}	~10,000 cd m^{-2}	30 s @ 4 V	Ref.23
$CsPbBr_3$-$ZnBr_2$ QDs	518 nm	16.48%	76,940 cd m^{-2}	~2,000 cd m^{-2}	136 min @ 0.6 mA cm^{-2}	Ref.24
$CsPbI_3$ QDs	653 nm	21.3%	500 cd m^{-2}	~70 cd m^{-2}	5 min @ 1.25 mA cm^{-2}	Ref.25
$CsPbI_3$ QDs	682 nm	15.1%	2,202 cd m^{-2}	-	--	Ref.26
CF_3KO_3S-*p*-FPEA QWs	526 nm	20.36%	82,480 cd m^{-2}	~ 47,600 cd m^{-2}	358 min @ 100 cd m^{-2}<br>6.5 min @ 10,000 cd m^{-2}	This work

Supplementary Method 1 | Determine the Photo-generated Carrier Density (N_0).

The initially photo-generated carrier density (N_0) of the perovskite can be evaluated by following equation[27-29]:

$$N_0 = \frac{E\lambda\alpha(\lambda)}{hcA_{eff.}}(1 - R_{pump}) \tag{12}$$

where E is the energy of the excited pump pulse; λ, $\alpha(\lambda)$ and R_{pump} represents the wave length, absorption coefficient and reflectivity of the excited pump pulse, respectively. $A_{eff.}$ is the light spot effective area.

The value of absorption coefficient ($\alpha(\lambda)$) can be obtained from UV-vis measurements and calculated as follows[27-29]:

$$\alpha(\lambda) = -\frac{1}{l} ln \frac{T}{1 - R} \tag{13}$$

where T, R and l is the transmittance, reflectivity and thickness of the perovskite film, respectively. Kramers-Kronig relationship is also a method to obtain the precise absorption coefficient. However, the influence of the quartz substrate significantly increases the difficulty of fitting the absorption coefficient of the film sample.

For the TA measurements, the effective area ($A_{eff.}$) is the efficient overlay area of the pump and the probe beam, which can be defined as follows[27-29]:

$$A_{eff.} = \frac{\pi}{\ln(2)}\left(FWHM_{pump}^2 + FWHM_{probe}^2\right) \tag{14}$$

Supplementary Note 1 | Energy transfer and recombination kinetics in quasi-2D perovskites. Decay kinetics of each ground state bleach are extracted from TA spectra (Supplementary Figures 6-8). The decay kinetics in both <*n*> = 4 quasi-2D perovskite films show obviously faster charge injection processes from donor to acceptor domains than that in <*n*> = 3 analogs (Supplementary Table 3). Thus, more-graded energy landscape within <*n*> = 4 quasi-2D perovskite films leads to a more efficient energy transfer, facilitating the radiative recombination.

In addition, the compromise between trap-assisted and exciton recombination is also a crucial issue for high PLQYs. Compared with <*n*> = 4 perovskite films, <*n*> = 5 analogs display almost 2-times higher density of defect state (Supplementary Figures 14 and 19). Thus, a mass of defect densities is inevitably introduced with the increasing proportion of large *n*-value domain (Supplementary Figures 6-8), leading to faster trap-assisted recombination rates in perovskite films[27].

Meanwhile, the exciton binding energy (E_b) is largely reduced as the <*n*>-value increased (Fig. 1g), leading to reduced exciton recombination rates. Thus, the synergy between the faster trap-assisted recombination rates and slower exciton recombination rates leads a significant decline for <*n*> = 5 perovskite films' PLQY.

In conclusion, we confirm that the more-graded energy landscape in both <*n*> = 4 quasi-2D perovskite films is the foundation for their high PLQYs, which ensures a more efficient energy transfer process and higher radiative recombination proportion.

Supplementary Note 2 | Auger recombination and emission behavior in quasi-2D perovskite films (<*n*> = 5) with large *n* domains. We obtained the high <*n*>-value quasi-2D perovskite films (<*n*> = 5) by simply modifying the stoichiometry (Supplementary Table 1). As expected, compared with <*n*> = 4 analogy, the flatted energy landscape composed by high *n*-value domain (*n* > 5) is largely increased, leading to an increased recombination center (Supplementary Figure 8).

It has been reported that Auger recombination can be suppressed by increasing the width of lower-bandgap quantum wells[30]. Furthermore, the exciton binding energy (E_b) of the perovskite film could be sequentially reduced with <*n*>-value increasing (Fig. 1g). Thus, compared with <*n*> = 4 analogies, higher thresholds for PLQY declining are found in both CF_3KO_3S treated <*n*> = 5 perovskite films. These results demonstrate that simply increasing the high *n* domain is do an effective approach for reducing the PLQY declining.

It is worth mentioning that, we found that the maximal PLQYs for <*n*> = 5 perovskite films were significantly sacrificed, even though after passivation engineering by CF_3KO_3S additive (Supplementary Figure 20). SCLC techniques are then employed to evaluate the defect state density. In particularly, compared with <*n*> = 4 perovskite films, <*n*> = 5 analogies display almost 2-times higher density of defect state (Supplementary Figure 19). These results clearly indicate that a mass of defect states is inevitably introduced with the increasing proportion of large *n*-value domain (Supplementary Figure 8), resulting in faster trap-assisted recombination rates[27].

TRPL measurements and TA kinetics are also employed to evaluate the recombination dynamics (Supplementary Figures 21, 22 and Supplementary Table 5). As shown, the Auger recombination rates display an obvious decline compared with <*n*> = 4 analogies, consistent well with the previous reports[30]. However, the k_1 of CF_3KO_3S-PEA (<*n*> = 5) also displays a significant decline due to the reduced E_b. The k_1 of CF_3KO_3S-*p*-FPEA (<*n*> = 5) increases probably due to the enhanced trap-assistant recombination[27]. The extracted kinetics are consistent well with the experimental results.

In brief, we confirm that the *n*-value domain distribution within PEA and *p*-FPEA perovskite films are similar; and the reduction of E_b enabled by introducing high-polarizable organic cation is the main reason for the suppressed Auger recombination. Furthermore, we confirm that simply increasing the high *n*-value domain can inevitably introduce a mass of defect states and sacrifice the PLQYs, though it is do an effective approach for reducing the Auger recombination.

Supplementary Note 3 | Efficiency roll-off in PeLEDs. Firstly, the trend of current density dependent EQE curves for different quasi-2D PeLEDs are consistent well with that of the corresponding films' PLQYs as a function of carrier density (Figs. 2d, 3c and 4d). This result undoubtedly demonstrates that reducing the Auger recombination rate can effectively suppress the efficiency roll-off of the operating devices[30,31].

It should be pointed out that, it is difficult to precisely correlate the charge-carrier density in the perovskite film produced by laser pulse with that in the LED device produced by continuous charge injection[7]. As well known, in addition to the Auger recombination rate of active materials, efficiency roll-off is attributed by several factors, such as leakage current, imbalanced charge injection, Joule heating, etc[32].

SEM images confirm the uniform and dense morphology of perovskite films (Supplementary Figure 25), demonstrating that the leakage current between charge transport layers is largely inhibited. Furthermore, the extremely low current density ($< 10^{-3}$ mA cm^{-2}) at ohmic response reconfirms the ignorable leakage current (Fig. 4b).

Meanwhile, we also qualitatively evaluate the charge injection through the hole- and electron-only device (Supplementary Figure 23)[11]. The current density between these devices is basically in the same magnitude under operating voltage range, demonstrating the balanced charge injection.

In addition, Joule heating is a crucial factor for devices' efficiency roll-off [33]. We noticed that the hole mobilities of the PEA and *p*-FPEA quasi-2D perovskite are only around 10^{-4} - 10^{-3} cm^2 V^{-1} s^{-1} (Supplementary Figure 14), which are four order of magnitude lower than 3D analogue[34]. Thus, low conductivity within quasi-2D perovskite films is a reasonable cause for the Joule heating, which aggravated the efficiency roll-off.

The low charge mobility as well as the conductivity can be attributed to two mean reasons: i) Presence of insulative large organic cation within quasi-2D perovskite films. ii) High charge injection barrier induced by the large bandgap of the low *n*-value domain within Br-based quasi-2D perovskites. Synergistically, the

threshold of our green-emission quasi-2D PeLEDs is slightly lower than that in green-emission 3D and near-infrared-emission quasi-2D PeLEDs[7,30].

In conclusion, we have obtained PeLEDs with higher efficiency based on two-dimensional perovskite materials. However, additional efforts on material science and device engineering are still required for further suppressing the efficiency roll-off and increasing the brightness.

Supplementary References

1. Hangleiter, A. & Häcker, R. Enhancement of band-to-band Auger recombination by electron-hole correlations. *Phys. Rev. Lett.* **65**, 215-218 (1990).
2. Ishihara, T., Takahashi, J. & Goto, T. Exciton state in two-dimensional perovskite semiconductor $(C_{10}H_{21}NH_3)_2PbI_4$. *Solid State Commun.* **69**, 933-936 (1989).
3. Hong, X., Ishihara, T., & Nurmikko. A. V. Dielectric confinement effect on excitons in PbI_4-based layered semiconductors. *Phys. Rev. B* **45**, 6961-6964 (1992).
4. Yuan, M. *et al.* Perovskite energy funnels for efficient light-emitting diodes. *Nat. Nanotechnol.* **11**, 872-877 (2016).
5. Quan, L. et al. Tailoring the energy landscape in quasi-2D halide perovskites enables efficient green-light emission. *Nano Lett.* **17**, 3701-3709 (2017).
6. Xing, G., *et al.* Transcending the slow bimolecular recombination in lead-halide perovskites for electroluminescence. *Nat. Commun.* **8**, 14558 (2017).
7. Chen, Z., *et al.* Recombination dynamics study on nanostructured perovskite light-emitting devices. *Adv. Mater.* 1801370 (2018).
8. Shi, D. *et al.* Low trap-state density and long carrier diffusion in organolead trihalide perovskite single crystals. *Science* **347**, 519-522 (2015).
9. Jiang, Y. *et al.* Spectra stable blue perovskite light-emitting diodes. *Nat. Commun.* **10**, 1868 (2019).
10. Wehrenfennig, C., *et al.* High charge carrier mobilities and lifetimes in organolead trihalide perovskites. *Adv. Mater.* **26**, 1584-1589 (2013).
11. Liu, X., *et al.* Metal halide perovskites for light-emitting diodes. *Nat. Mater.* (2020). https://doi.org/10.1038/s41563-020-0784-7.
12. Quan, L., et al. Edge stabilization in reduced-dimensional perovskites. *Nat. Commun.* **11**, 170 (2020).
13. Liu, Y., *et al.* Efficient blue light-emitting diodes based on quantum-confined bromide perovskite nanostructures. *Nat. Photonics* **13**, 760-764 (2019).
14. Ban, M., *et al.* Solution-processed perovskite light emitting diodes with

efficiency exceeding 15% through additive-controlled nanostructure tailoring. *Nat. Commun.* **9**, 3892 (2018).

15. Wu, C., *et al.* Alternative type two-dimensional-three-dimensional lead halide perovskite with inorganic sodium ions as a spacer for high-performance light-emitting diodes. *ACS Nano* **13**, 1645-1654 (2019).
16. Yang, X., *et al.* Effects of organic cations on the structure and performance of quasi-two-dimensional perovskite-based light-emitting diodes. *J. Phys. Chem. Lett.* **10**, 2892-2897 (2019).
17. Wang, H., *et al.* Trifluoroacetate induced small-grained $CsPbBr_3$ perovskite films result in efficient and stable light-emitting devices. *Nat. Commun.* **10,** 665 (2019).
18. Wu, T., *et al.* High-performance perovskite light-emitting diode with enhanced operational stability using lithium halide passivation. *Angew. Chem. Int. Edit.* **59**, 2-9 (2019).
19. Lin, K., *et al.* Perovskite light-emitting diodes with external quantum efficiency exceeding 20 per cent. *Nature* **562,** 245-248 (2018).
20. Fang, Z., *et al.* Dual passivation of perovskite defects for light-emitting diodes with external quantum efficiency exceeding 20%. *Adv. Funct. Mater.* 1909754 (2020).
21. Zhang, X, *et al.* Highly efficient light emitting diodes based on in situ fabricated $FAPbI_3$ nanocrystals: solvent effects of on-chip crystallization. *Adv. Opt. Mater.* **7,** 1900774 (2019).
22. Zhao, L., Lee, K. M., Roh, K., Khan, S. U. Z., & Rand, B. P. Improved outcoupling efficiency and stability of perovskite light-emitting diodes using thin emitting layers. *Adv. Mater.* 2805836 (2018).
23. Han, D., *et al.* Efficient light-emitting diodes based on in situ fabricated $FAPbBr_3$ nanocrystals: the enhancing role of ligand-assisted reprecipitation process. *ACS Nano* **12**, 8808-8816 (2018).
24. Song, J., *et al.* Organic-inorganic hybrid passivation enables perovskite QLEDs with an EQE of 16.48%. *Adv. Mater.* **30,** 1805409 (2018).
25. Takayuki, C., *et al.* Anion-exchange red perovskite quantum dots with

ammonium iodine salts for highly efficient light-emitting devices. *Nat. Photonics* **12**, 681-687 (2018).

26. Shen, X., *et al.* Zn-alloyed $CsPbI_3$ nanocrystals for highly efficient perovskite light-emitting devices. *Nano Lett.* **19**, 1552-1559 (2019).
27. Milot, R., *et al.* Radiative monomolecular recombination boosts amplified spontaneous emission in $HC(NH_2)_2SnI_3$ perovskite films. *J. Phys. Chem. Lett.* **7**, 4178-4184 (2016).
28. Song, T. *et al.* Importance of reducing vapor atmosphere in the fabrication of tin-based perovskite solar cells. *J. Am. Chem. Soc.* **139**, 836-842 (2017).
29. Wenger, B., *et al*. Consolidation of the optoelectronic properties of $CH_3NH_3PbBr_3$ perovskite single crystals. *Nat. Commun.* **8**, 590 (2017).
30. Zou, W., *et al.* Minimising efficiency roll-off in high-brightness perovskite light-emitting diodes. *Nat. Commun.* **9**, 608 (2018).
31. Yang, M., *et al.* Reduced efficiency roll-off and enhanced stability in perovskite light-emitting diodes with multiple quantum wells. *J. Phys. Chem. Lett.* **9** 2038-2042 (2018).
32. Kim, H., *et al.* Hybrid perovskite light emitting diodes under intense electrical excitation. *Nat. Commun.* **9**, 4993 (2018).
33. Zou, C., *et al.* Suppressing efficiency roll-off at high current densities for ultra-bright green perovskite light-emitting diodes. *ACS Nano* **14**, 6076-6086 (2020).
34. Saidaminov, M., *et al.* High-quality bulk hybrid perovskite single crystals within minutes by inverse temperature crystallization. *Nat. Commun.* **6**, 7586 (2015).
